# Influence of Microenvironmental Orchestration on Multicellular Lung Alveolar Organoid Development from Human Induced Pluripotent Stem Cells

**DOI:** 10.1007/s12015-024-10789-1

**Published:** 2024-10-17

**Authors:** Vedat Burak Ozan, Huijuan Wang, Akshay Akshay, Deepika Anand, Youssef Hibaoui, Anis Feki, Janine Gote-Schniering, Ali Hashemi Gheinani, Manfred Heller, Anne-Christine Uldry, Sophie Braga Lagache, Amiq Gazdhar, Thomas Geiser

**Affiliations:** 1 Department for Pulmonary Medicine, Allergology and Clinical Immunology, Inselspital, Bern University Hospital, University of Bern, Bern, Switzerland; 2 Lung Precision Medicine (LPM), Department for BioMedical Research (DBMR), University of Bern, Bern, Switzerland; 3 Department of Gynecology and Obstetrics, Cantonal Hospital Fribourg, Fribourg, Switzerland; 4 Graduate School for Cellular and Biomedical Sciences (GCB), University of Bern, Bern, Switzerland; 5 Functional Urology Research Group, Department for BioMedical Research (DBMR), University of Bern, Bern, Switzerland; 6 Department of Urology, Inselspital, Bern University Hospital, University of Bern, Bern, Switzerland; 7 Urological Diseases Research Center, Boston Children’s Hospital, Boston, MA USA; 8 Department of Surgery, Harvard Medical School, Boston, MA USA; 9 Broad Institute of MIT and Harvard, Cambridge, MA USA; 10 Department of Rheumatology and Immunology Inselspital, Bern University Hospital, University of Bern, Bern, Switzerland; 11 Proteomics and Mass Spectrometry Core Facility, Department for BioMedical Research (DBMR), University of Bern, Bern, Switzerland

**Keywords:** 3D organoids, Lung organoids, Induced pluripotent stem cells, Lung proteomics, Multicellular lung organoid

## Abstract

Induced pluripotent stem cells (iPSCs) have emerged as promising in vitro tools, providing a robust system for disease modelling and facilitating drug screening. Human iPSCs have been successfully differentiated into lung cells and three-dimensional lung spheroids or organoids. The lung is a multicellular complex organ that develops under the symphonic influence of the microenvironment. Here, we hypothesize that the generation of lung organoids in a controlled microenvironment (cmO) (oxygen and pressure) yields multicellular organoids with architectural complexity resembling the lung alveoli. iPSCs were differentiated into mature lung organoids following a stepwise protocol in an oxygen and pressure-controlled microenvironment. The organoids developed in the controlled microenvironment displayed complex alveolar architecture and stained for SFTPC, PDPN, and KRT5, indicating the presence of alveolar epithelial type II and type I cells, as well as basal cells. Moreover, gene and protein expression levels were also increased in the cmO. Furthermore, pathway analysis of proteomics revealed upregulation of lung development-specific pathways in the cmO compared to those growing in normal culture conditions. In summary, by using a controlled microenvironment, we established a complex multicellular lung organoid derived from iPSCs as a novel cellular model to study lung alveolar biology in both lung health and disease.

**Graphical Abstract:**

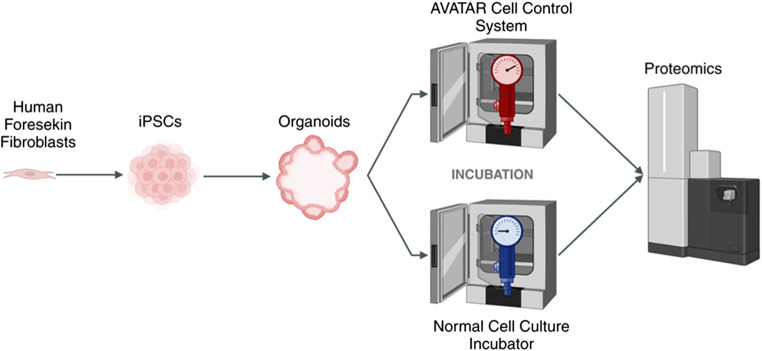

**Supplementary Information:**

The online version contains supplementary material available at 10.1007/s12015-024-10789-1.

## Introduction

The development of the lung is an intricate process of organogenesis, representing a complex interplay between a mosaic of molecular signals and the dynamic influence of the microenvironment with physical factors like oxygen and pressure [1, 2]. This complexity is underscored by the dynamic interplay between a diverse array of molecular signals and the profound influence of the microenvironment. Within this microenvironment, key factors such as the embryo’s genetic makeup, maternal environment, and the physical conditions within the embryo play pivotal roles in shaping lung development [1, 3]. The microenvironment plays a critical role in directing cell differentiation, proliferation, and morphogenesis, and ensuring the proper development of the airways and alveoli, the tiny air sacs where gas exchange occurs [4].

In lung development, two pivotal elements of the microenvironment play crucial roles: (i) oxygen concentration [5, 6] and (ii) pressure [7]. These elements, varying across different stages of development, are regulated by a diverse array of factors over time. Therefore, it is important to study the effects of these two factors on the development of the distal lung alveolar space. Historically, mouse models have been the cornerstone of developmental studies [8]. Recently, however, there has been a shift towards using progenitor cells derived from human fetal tissue. Despite the promises this approach holds, acquiring early fetal tissue poses significant technical and ethical challenges [9, 10].

Induced pluripotent stem cells (iPSC) have emerged as a very helpful tool to generate different cell types to understand development and disease pathophysiology and drug screening [11]. In recent years, the concept of developing 3D organoids, mini-organs grown in a dish, has emerged as pivotal entities for scientific research [12]. These are miniature, self-organizing structures, bearing a striking resemblance to organs and contribute in our approach to understand intricate biological processes [13]. Notably, these microscale models serve as invaluable tools for investigating organ development, closely replicating physiological functions within a controlled laboratory environment [14].

Our current study aims to elucidate the impact of the microenvironment, particularly oxygen concentration and pressure, on the development of distal alveolar organoids. Following a stepwise protocol for lung organoid generation by differentiating iPSCs, we have observed notable enhancements in gene and protein expression levels of key lung development regulators. Additionally, there was a distinct improvement in morphology and an increase in branching patterns when differentiating iPSCs in a dynamically controlled microenvironment, leading to the formation of multicellular alveolar lung organoids. These findings underscore the critical role of microenvironmental factors in lung organogenesis and open new avenues for therapeutic exploration.

## Results

### Experimental Design

**Fig. 1:**
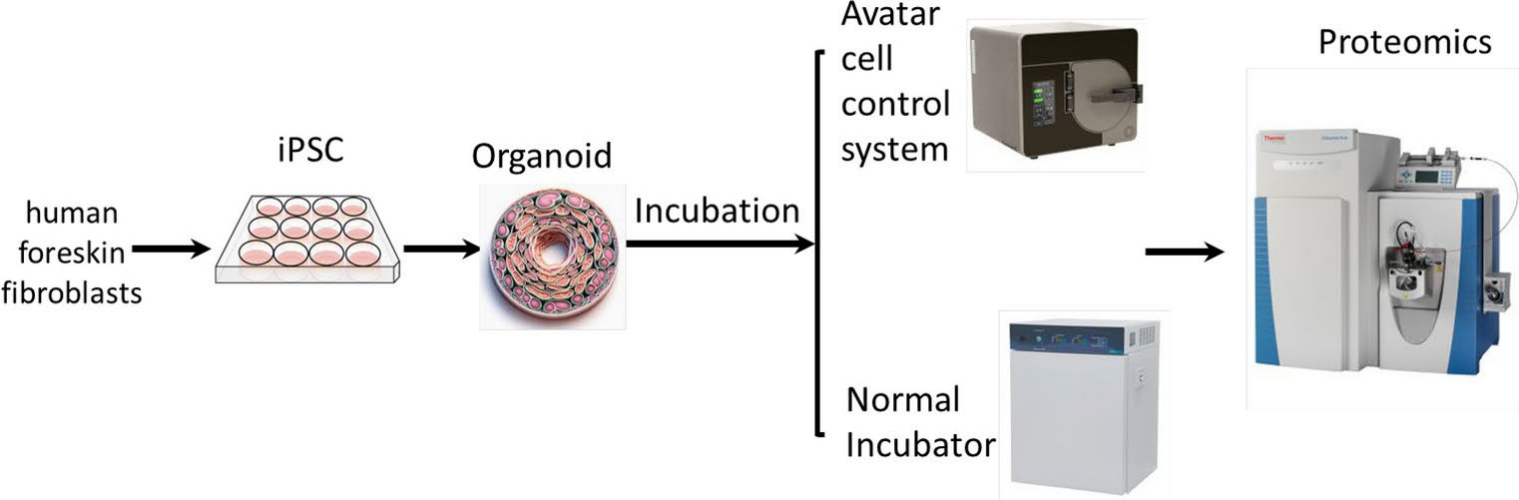
Schematic of the project outline. The iPSCs were differentiated to mature lung organoids either in the normal incubator at 37ºC, 5% CO2 and atmospheric pressure or in a controlled microenvironment using the commercially available Avatar cell control system

Graphic representation of the experimental design and the methods applied (Fig. 1).

### Increased Cell Numbers and Gene Expression During the Differentiation in the Controlled Microenvironment Organoids (cmO)

**Fig. 2:**
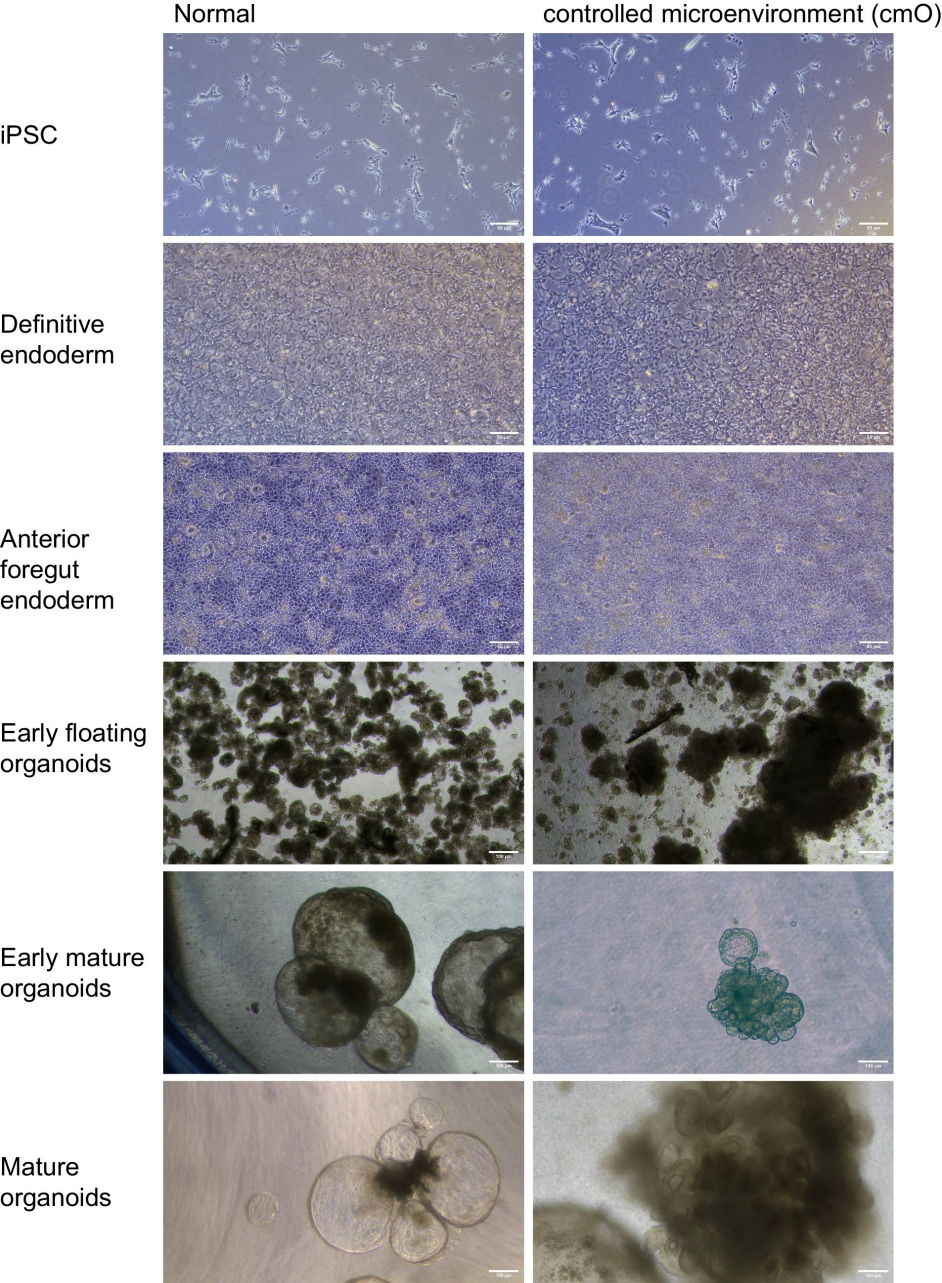
Brightfield microscopy images of all the stages of differentiation in both conditions. iPSCs to definitive endoderm, anterior foregut endoderm, early floating organoids (Day 10 from the beginning of differentiation), early mature organoids (Day 25 from the beginning of differentiation), late mature organoids (Day 40 from the beginning of differentiation)

Bright field images of the cells at different stages of differentiation from iPSCs to mature organoids in normal condition (left) and cmO (right). Induced pluripotent stem cells were grown at low density to start differentiation. The cells were then directed towards definitive endoderm, cell proliferation was increased and the cells grew as monolayer to definitive endoderm. After 3 days the cells were directed towards anterior foregut endoderm that led to increased cell density with inter spread areas of clustered cells and changed cell morphology. Interestingly, there was no observable difference noted in the two conditions. Furthermore, early organoids with rounded structures were observed in normal condition, however in cmO the organoids had denser structures when grown in low attachment plate. These early organoids were put on Matrigel sandwich for further maturation. Matured lung organoids in Matrigel sandwich after 40 days demonstrated varied morphologies like rounded alveolospheres with dense expansion tips in orgnaoids growing in normal condition. However, more complex branching structures with dense cells were observed in the organoids growing in cmO. (Fig. 2). Cells in all stages were analysed for surface marker and intracellular marker expression by imaging flow cytometry and the data is presented as the percentage of live cells expressing the measured marker and is compared between organoids growing in normal condition vs cmO. Definitive endoderm (3a) Nkx2.1 (44 ± 6.8 vs 65 ± 3.7), (3b) CXCR4 (48 ± 6 vs 70 ± 5.3), (3c) cKit (45 ± 10 vs 63 ± 5.4), (3d) CXCR4/cKit (45 ± 10 vs 71 ± 2.6). Anterior foregut endoderm (3e) Epcam (65 ± 2.3 vs 66 ± 4.3), (3f) Nkx2.1 (63 ± 3.1 vs 92 ± 0.3), (3 g) Pax9 (3.4 ± 0.31vs 4.7 ± 0.34), (3 h) Sox9 (9.2 ± 0.36 vs 6.0 ± 0.12). Early lung organoids (3i) Epcam (54 ± 3.5 vs54 ± 0.1), (3j) Sox9 (54 ± 3.5 vs 43 ± 4.3), (3 k) Nkx2.1 (17 ± 1 vs 32 ± 14), (3 l) Epcam/SFTPB (26 ± 2.3 vs 19 ± 1), (3 m) Epcam/SFTPC (26 ± 1vs 80 ± 1.6). Mature lung organoids (3n) Epcam (79 ± 1.6 vs 80 ± 9.2), (3o) Sox9 (14 ± 3.1vs 13 ± 5.1), (3p) Nkx2.1 (4.7 ± 0.08 vs 5.8 ± 1.2), (3q) Epcam/SFTPB (18 ± 2.6 vs 73 ± 12), (3r) Epcam/SFTPC (28 ± 4 vs 82 ± 4.3).

**Fig. 3:**
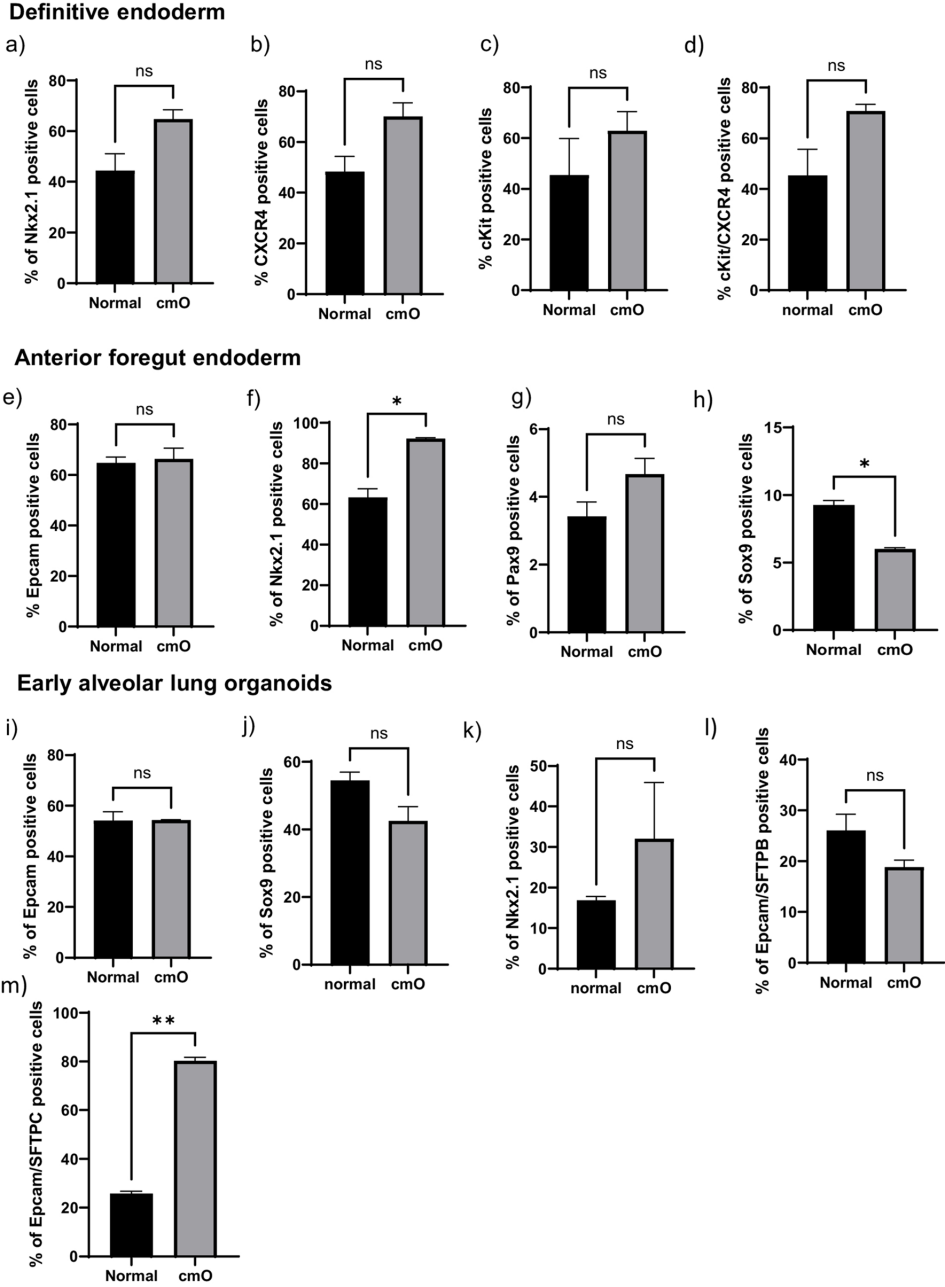

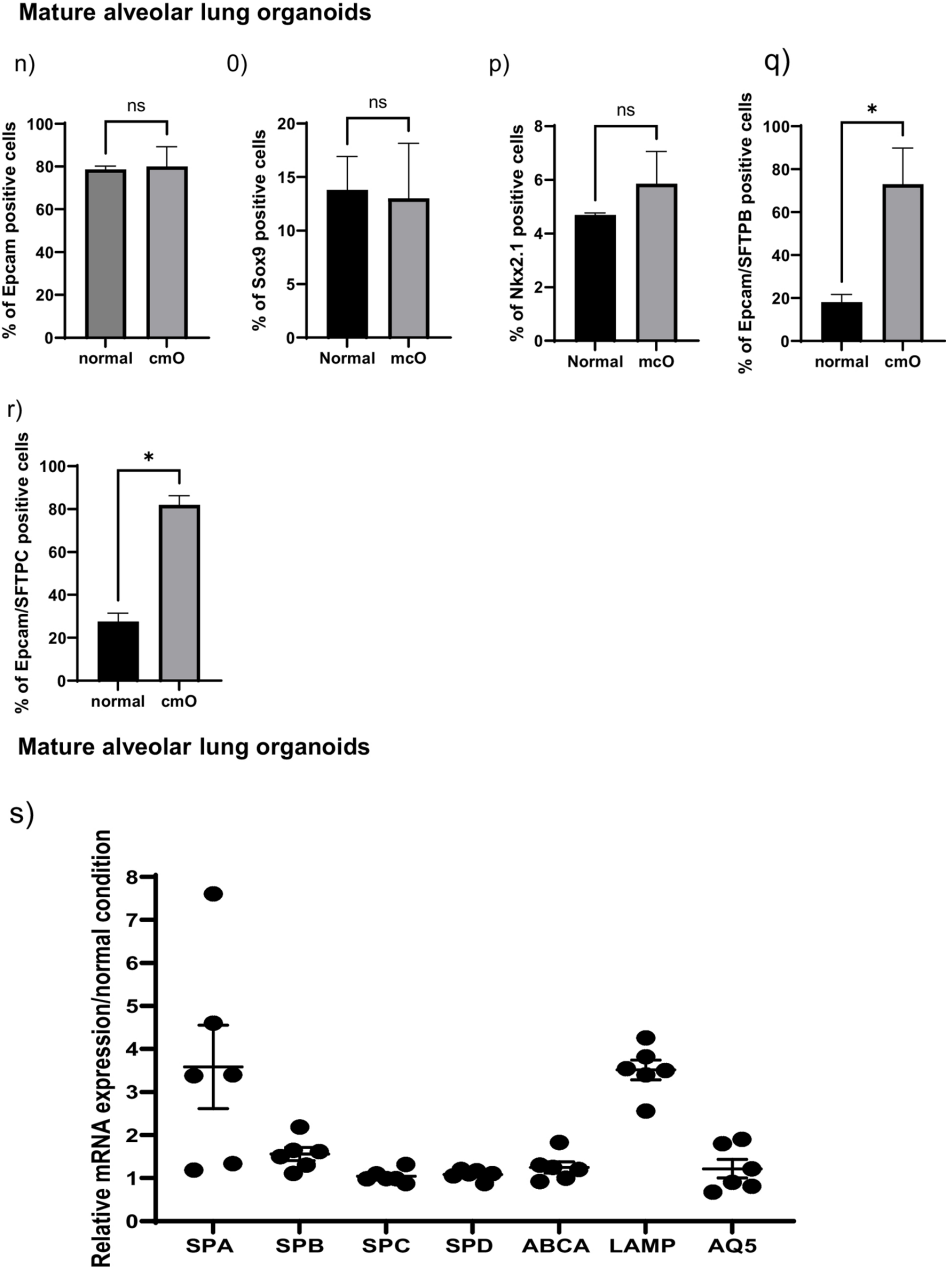
Flow cytometry analysis performed at different stages of the differentiation for definitive endoderm NKX2.1(**a**) CXCR4 (**b**). For anterior foregut endoderm, NKX2.1(**c**), EpCAM (**d**), PAX9 (**e**). To characterize early alveolar organoids NKX2.1 (**f**), EpCAM (**g**), SFTPB (**h**), SFTPC (**i**), double positive for SFTPB and SFTPC (**j**), SOX9 (**k**). For mature alveolar lung organoids NKX2.1 (**l**), EpCAM (**m**), SFTPB (**n**), SFTPC (**o**), double positive SFTPB and SFTPC (**p**), SOX9 (**q**). Relative mRNA expression of markers of alveolar epithelial type I and epithelial type II cells. The data is presented as fold change in cmO normalized to organoids growing in normal conditions. For mRNA expression four transwell inserts were used for each condition. For flow cytometery experiments, four transwell inserts were pooled for early lung organoids and mature lung organoids to obtained high cell number the experiments were repeated twice and the data is presented as mean ± SEM

Relative mRNA expression showed increased relative mRNA expression of SFTPA (3.6 ± 0.97), SFTPB (1.6 ± 0.15), SFTPC (1 ± 0.06), SFTPD (1.1 ± 0.04), ABCA (1.2 ± 0.13), LAMP (3.5 ± 0.23), AQ5 (1.20 ± 0.21) in cmO compared to normal organoids (Fig. 3s).

### Defined Architecture with Branching in cmO Compared to Normal Organoids

**Fig. 4:**
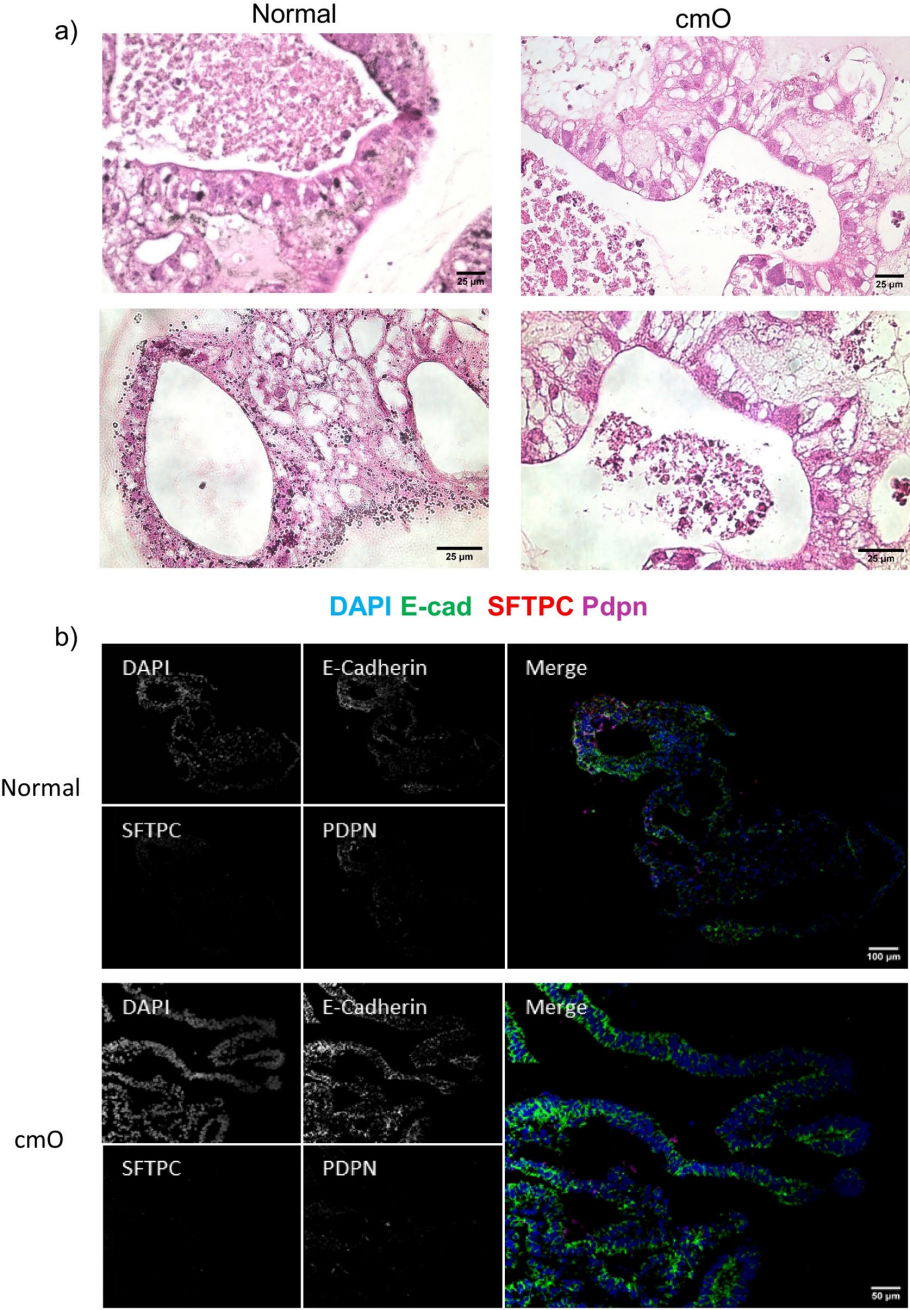

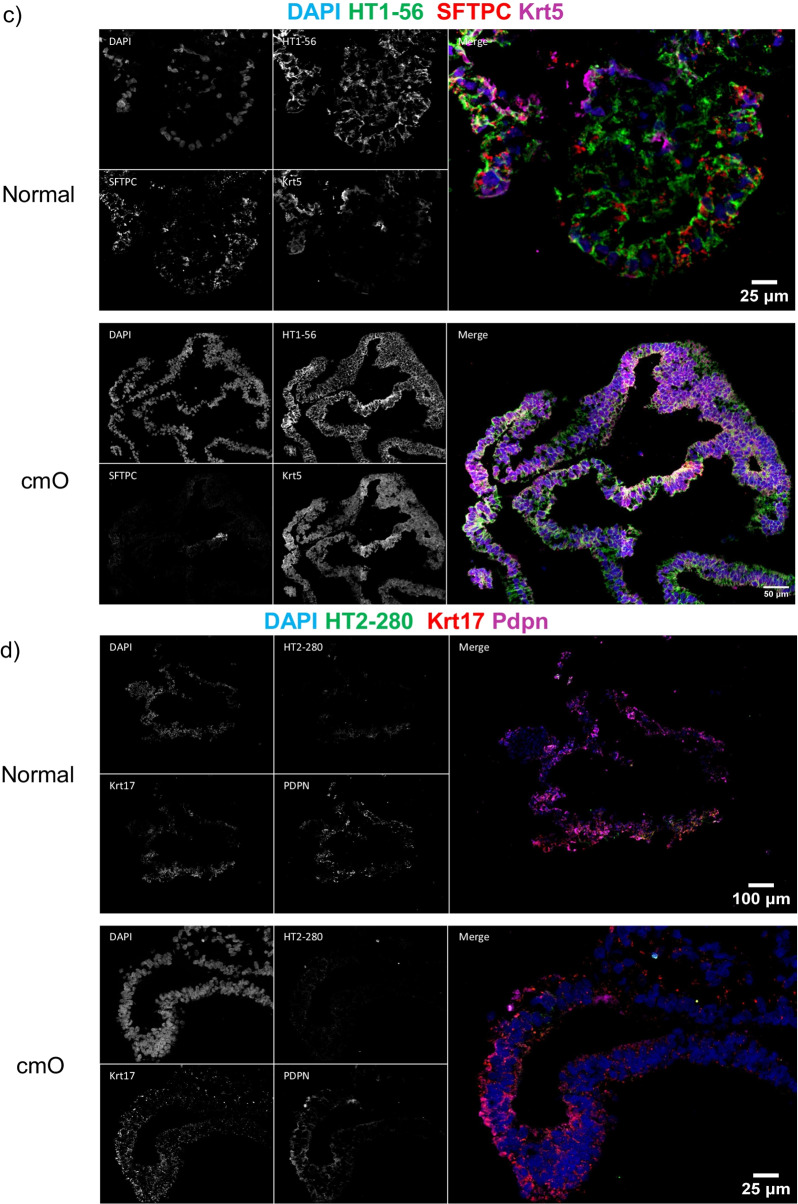

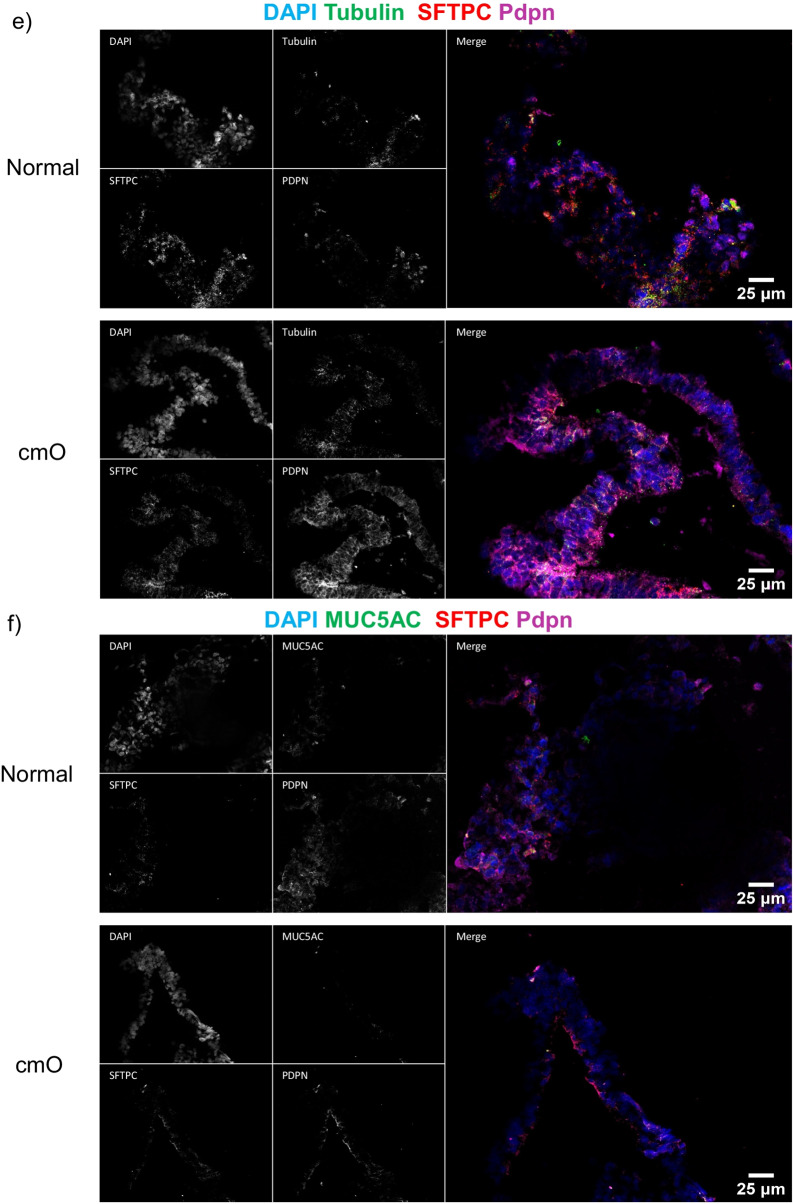
(**a**) Histological analysis of organoids was done, and haematoxylin and eosin (H&E) staining was performed. Immunofluorescence imaging was performed to stain the sections for various markers of alveolar epithelial cell markers and for markers of alveolar stem cells: (**b**) DAPI, E-cadherin, SPC and PDPN, (**c**) DAPI, HT1-56, SPC, KRT5, (**d**) DAPI, HT2-280, KRT17, PDPN, (**e**) DAPI, Tubulin, SPC, PDPN, (**f**) DAPI, MUC5AC, SPC, PDPN. The individual images of each staining are represented. Two transwell inserts were fixed for each condition and immunofluoroscence imaging was performed

**Fig. 5:**
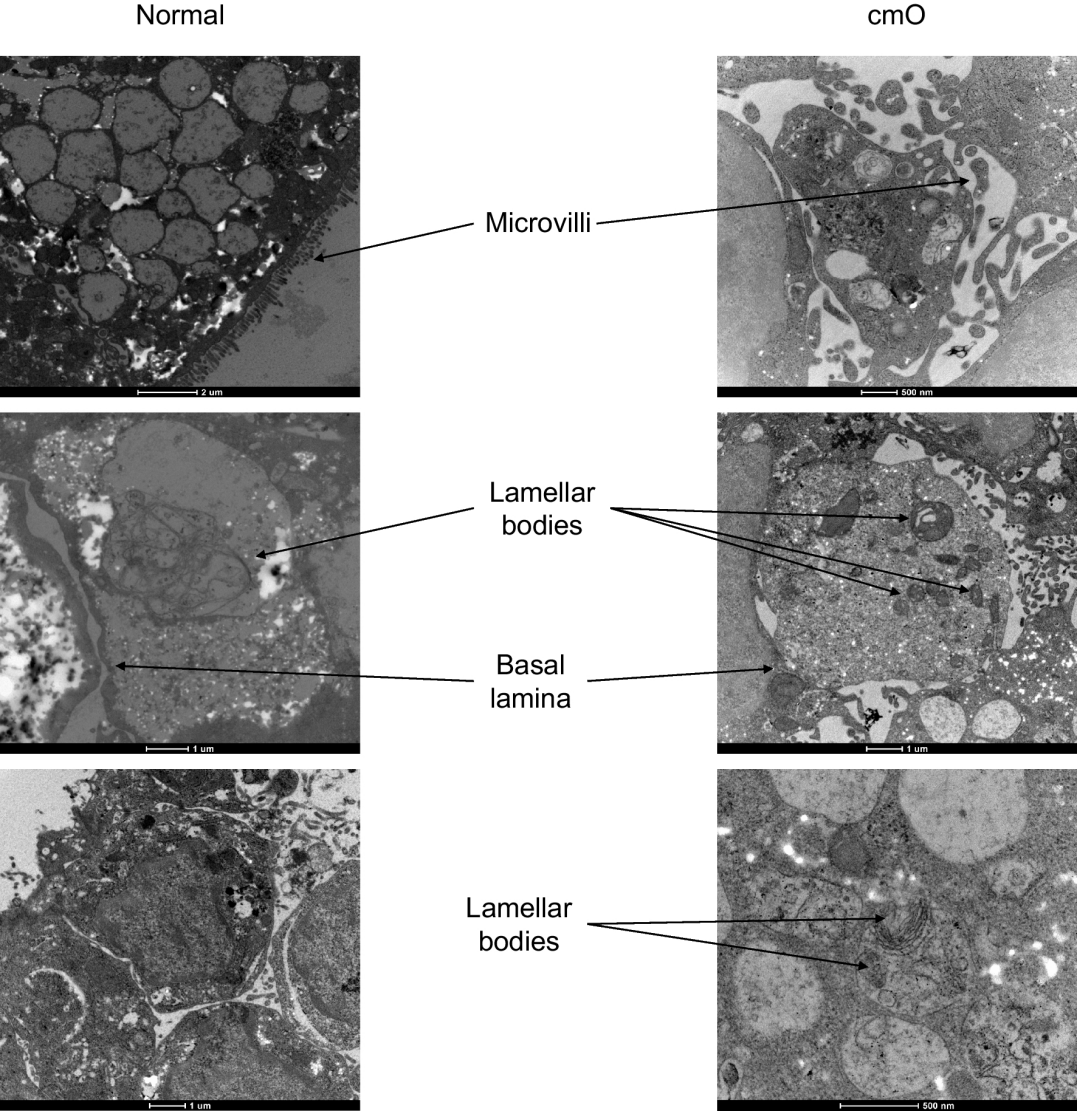
Electron microscopy images of normal and cmO organoids. Normal organoids showing microvilli and loose lamellar bodies, and cmO showing microvilli and well structured lamellar bodies. Two transwell inserts were fixed for each condition and electron microscopy was performed

The mature organoids were fixed in paraformaldehyde and stained for haematoxylin and eosin (H&E). The cmO exhibited more defined branching and cellular distribution resembling the architecture of the distal lung, and the distribution of cells was organized similarly to that observed in the distal lung (Fig. 4a). Immunostaining with markers of epithelial cells, SFTPC, HT1-56, HT2-280, E-cadherin, PDPN (Fig. 4b-f) confirmed a cellular distribution akin to that in the distal lung. Notably, cmO also stained for KRT 5 (Fig. 4c) showing the presence of basal cells in the organoids. Additionally, transmission electron microscopy (TEM) revealed the presence of lamellar bodies, a distinctive feature of alveolar epithelial type II cells in both the organoids (Fig. 5). Interestingly, more lamellar bodies with more condensed structure were seen in cmO compared to the organoids growing in normal conditions. Furthermore, more organized and structured microvilli were also seen in the cmO. (Fig. 5).

### Proteomic Analysis Reveals Environmental Impact on Protein Regulation in Organoids

**Fig. 6:**
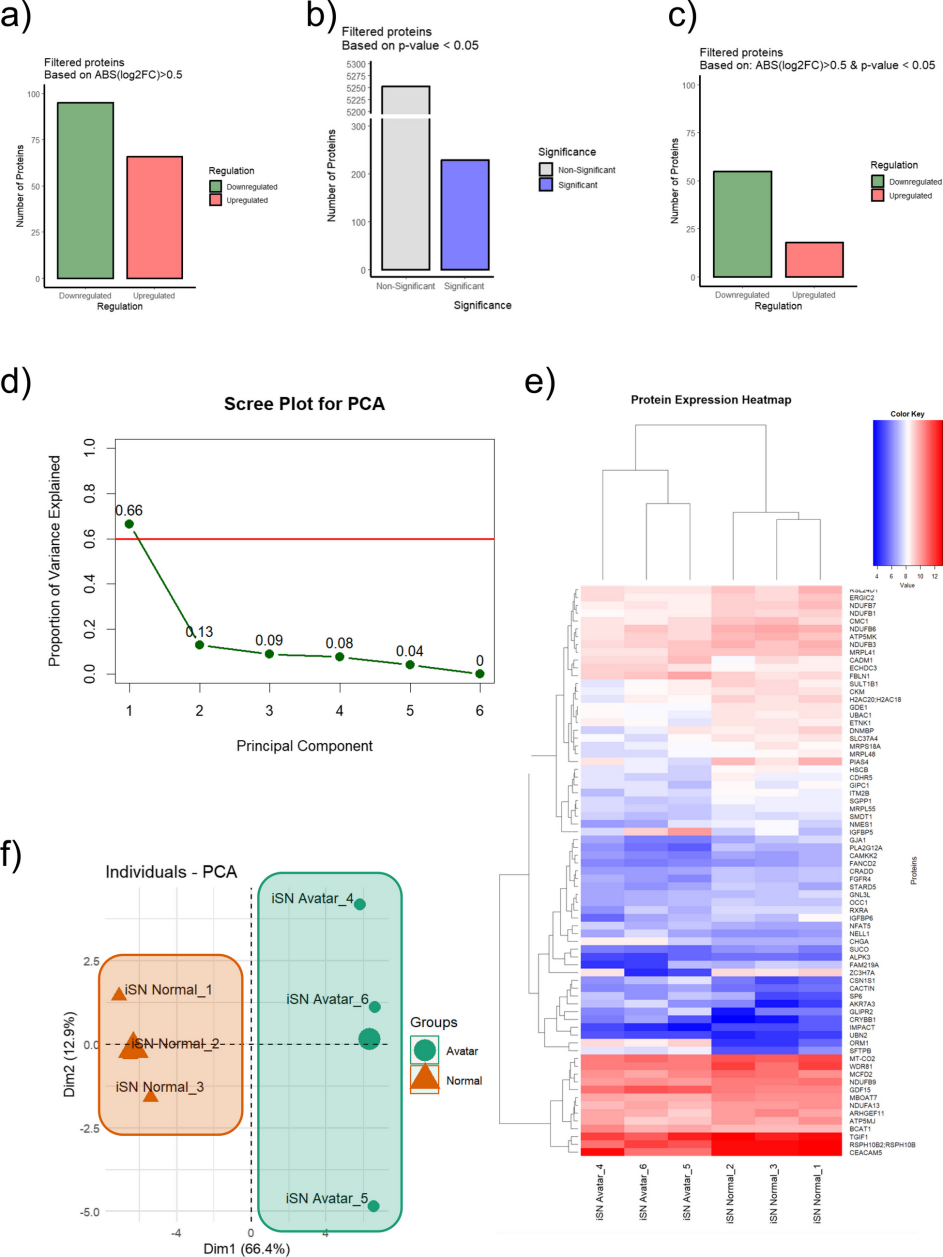
(**a**) Bar graphs showing the number of proteins filtered based on absolute log2(fold change) > 0.5 with a delineation between downregulated and upregulated proteins. (**b**) Bar graphs representing proteins filtered based on *p*-value < 0.05. (**c**) Bar graphs representing proteins filtered based on both absolute log2(fold change) > 0.5 and *p*-value < 0.05, categorized by significance and regulation. The colour coding in the graphs serves to visually distinguish between downregulated (green), upregulated (red), non-significant (grey), and significant (blue) proteins, based on their expression changes and statistical relevance. (**d**) Scree plot illustrating the proportion of variance explained by each principal component in a PCA analysis. (**e**) Two-dimensional PCA plot of proteomic analysis results, contrasting organoids cultured in a controlled microenvironment (cmO) within an Avatar incubator (green) with those grown under standard culture conditions (orange), showcasing the principal component distribution for Dim1 and Dim2 which captures the most significant variation in the data. (**f**) Heatmap with hierarchical clustering showing the expression patterns of proteins across multiple samples, with a colour key indicating expression levels from low (blue) to high (red). Each row represents a unique protein, and each column corresponds to a sample. Hierarchical clustering using the Euclidean distance method has been applied to both rows and columns to group proteins and samples with similar expression patterns, respectively. Three transwell inserts were taken for proteomic analysis from each condition (n = 3)

Comprehensive proteomic analysis revealed that the controlled microenvironment has a significant impact on the regulation of proteins. We observed a distinct difference in the number of proteins that were either upregulated or downregulated, with a substantial portion showing significant changes in expression levels when filtered by both an absolute log2 (fold change) > 0.5 and a* p*-value < 0.05 (Fig. 6a-c).

As it is depicted in Fig. 6a, proteins were categorized based on an absolute log2 fold change (log2FC) greater than 0.5, showing 95 proteins were downregulated (illustrated in green) and about 66 were upregulated (shown in red). Figure 6c focuses on proteins filtered by statistical significance, with a p-value threshold of less than 0.05, revealing approximately 5252 proteins as non-significant (grey bar) and around 229 as significant (blue bar) regardless of their fold change regulation. Figure 6d combines both the log2FC and p-value criteria, indicating nearly 55 proteins as downregulated and about 18 as upregulated when both conditions are met.

The scree plot based on the proteomics data provided a visual representation used to determine the number of principal components to retain in a principal component analysis (PCA) for proteomics data. The first principal component accounted for a substantial proportion of the variance (66%), indicating that it captures a significant amount of the information in the dataset (Fig. 6d). The second principal component accounts for an additional 13% of the variance, while subsequent components contribute progressively less, with the sixth component contributing virtually no additional information. Further analysis of the proteomic data through PCA demonstrated that the differentially regulated proteins could effectively separate the samples into distinct groups. This is evidenced by the scree plot in Fig. 6c and d, which shows the proportion of variance explained by each principal component, and the two-dimensional PCA plot in Figs. 5 and 6f, where the principal components that captured the most significant variation in the data were able to discriminate between the cmO and the standard culture conditions.

Moreover, hierarchical clustering of the samples, as shown in Fig. 6e, indicated that organoids cultured under standard conditions clustered tightly with one another, as did those from the controlled microenvironment. This pattern suggests that the distinct proteomic profiles are consistent within each group, emphasizing the reproducibility of the environmental effects on protein expression. The clustering, performed using the Euclidean distance method, reinforced the observation that the cmO can create a unique proteomic signature compared to the standard culture conditions, potentially underlying functional and phenotypic differences in organoid development.

### Proteomic Adaptations in Organoid Cultures: Differential Protein Expression and Cellular Responses to Controlled Incubation Environments

**Fig. 7:**
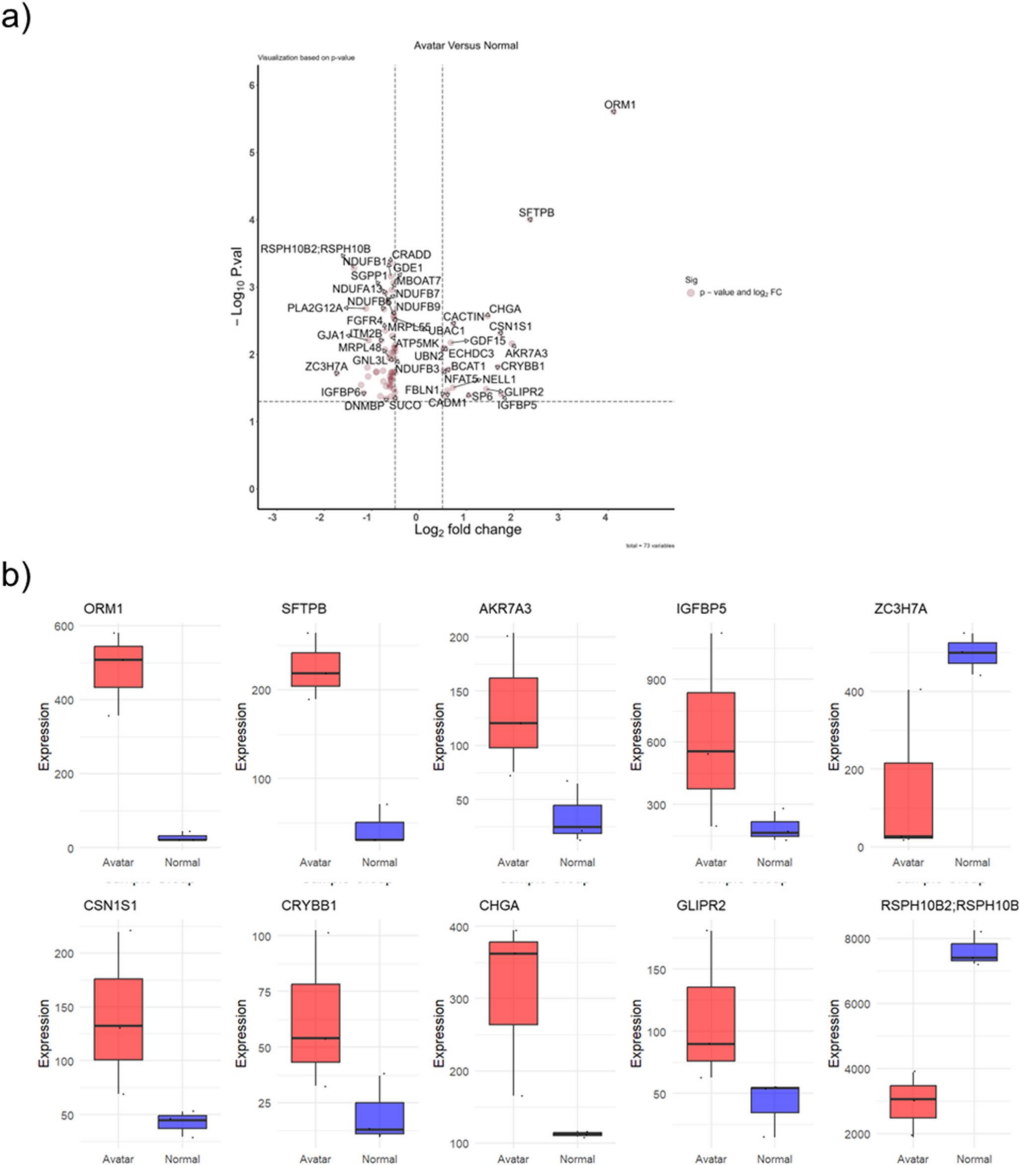
Differential Protein Expression in Organoid Cultures from Avatar and Normal Incubators. (**a**) a volcano plot displaying the log2 fold change versus the negative log of the p-value for proteins analysed. Proteins with a statistically significant upregulation in the Avatar incubator are shown to the right and those with downregulation to the left, with the degree of significance indicated by the height on the y-axis. (**b**) Boxplots for the top differentially expressed proteins, with the red boxes representing protein expression levels in organoids cultured in the Avatar incubator and the blue boxes indicating expression levels in organoids from the normal incubator. The central line in each box represents the median expression level, the box boundaries indicate the interquartile range (IQR), and the whiskers extend to 1.5 times the IQR. Outliers are represented as individual points

Assessing differential protein expression between organoids cultivated in the Avatar incubator and those grown under standard conditions using the volcano plot (Fig. 7a) we identified the most significantly regulated proteins, with “ORM1” markedly upregulated, indicating a pronounced response to the controlled microenvironment. Similarly, “SFTPB” emerges as another significantly upregulated protein, reinforcing the impact of the incubation conditions on the proteomic landscape. Conversely, “ZC3H7A” is noted as a downregulated protein, which might be indicative of repression in the expression within the controlled environment. The distribution and placement of these proteins on the volcano plot not only reflects their individual expression changes but also underscores the overall trend in protein regulation under varying incubation conditions, offering insightful cues into the proteomic adaptations that organoids undergo in response to environmental modifications.

Figure 7b features a series of boxplots that elucidate the regulation of specific proteins under varying incubation conditions, providing insights into their potential roles in cellular processes within organoids. The expression of ORM1, known for its involvement in the acute phase response and transport, is notably higher in organoids cultured in the Avatar incubator, suggesting an enhanced stress response or altered transport dynamics under controlled conditions. Similarly, SFTPB, which is crucial for alveolar stability and gaseous exchange, also shows elevated expression in the Avatar group, pointing towards a potential adaptation or maintenance mechanism in response to the incubation environment. Akr7A3, an oxidoreductase, presents with a modest upregulation in the Avatar incubator group, indicating possible shifts in oxidative stress management or metabolic processes. IGFBP5, associated with growth factor binding and cell migration, is another protein that exhibits a significant upregulation, which could reflect changes in growth signalling pathways or cellular dynamics. CSN1S1, involved in calcium phosphate transport, and CRYBB1, an eye lens protein with an unclear function in the lung, both display a lower expression in the Avatar group, raising questions about their roles under controlled incubation conditions.

CHGA, a precursor to several biologically active peptides, shows a pronounced increase in expression, suggesting alterations in the neuroendocrine signalling within the organoid cultures. GLIPR2, linked to the regulation of epithelial cell migration, also exhibits increased expression, hinting at changes in cellular movement or organization. RSPH10B2, essential for ciliary motility, and ZC3H7A, a regulator of miRNA biogenesis and RNA-binding, displays lower expression levels in the Avatar group, suggesting potential modifications in gene expression regulation and cellular structures.

The differential expression patterns of these proteins provide a window into the molecular adaptations organoids undergo in response to the controlled microenvironment of the Avatar incubator compared to standard conditions. The data imply a complex interplay of stress responses, metabolic adjustments, signalling alterations, and structural changes that could be foundational in understanding the impact of incubation environments on organoid development and function. List of top 10 regulated protein shown in Table 1.

**Table 1:** Protein expression

Protein	Biological Process	Expression in controlled microenvironment
ORM 1	Acute phase, Transport	High
SFTPB	alveolar stability, Gaseous exchange	High
Akr7A3	Oxidoreductase	High
IGFBP 5	Growth factor binding, cell migration	High
CSN1S1	Calcium phosphate transport	High
CRYBB1	Eye lens protein (function in lung not known)	High
CHGA	precursor to biologically active peptides; vasostatin, pancreastatin, and parastatin	High
GLIPR2	regulation of epithelial cell migration	Low
RSPH10B2	ciliary motility	Low
ZC3H7A	regulator of miRNA biogenesis, RNA-binding	Low

### Impact of Incubation Conditions on Organoid Respiratory Tract Morphogenesis: Integrated Pathway and Functional Genomics Analysis

**Fig. 8:**
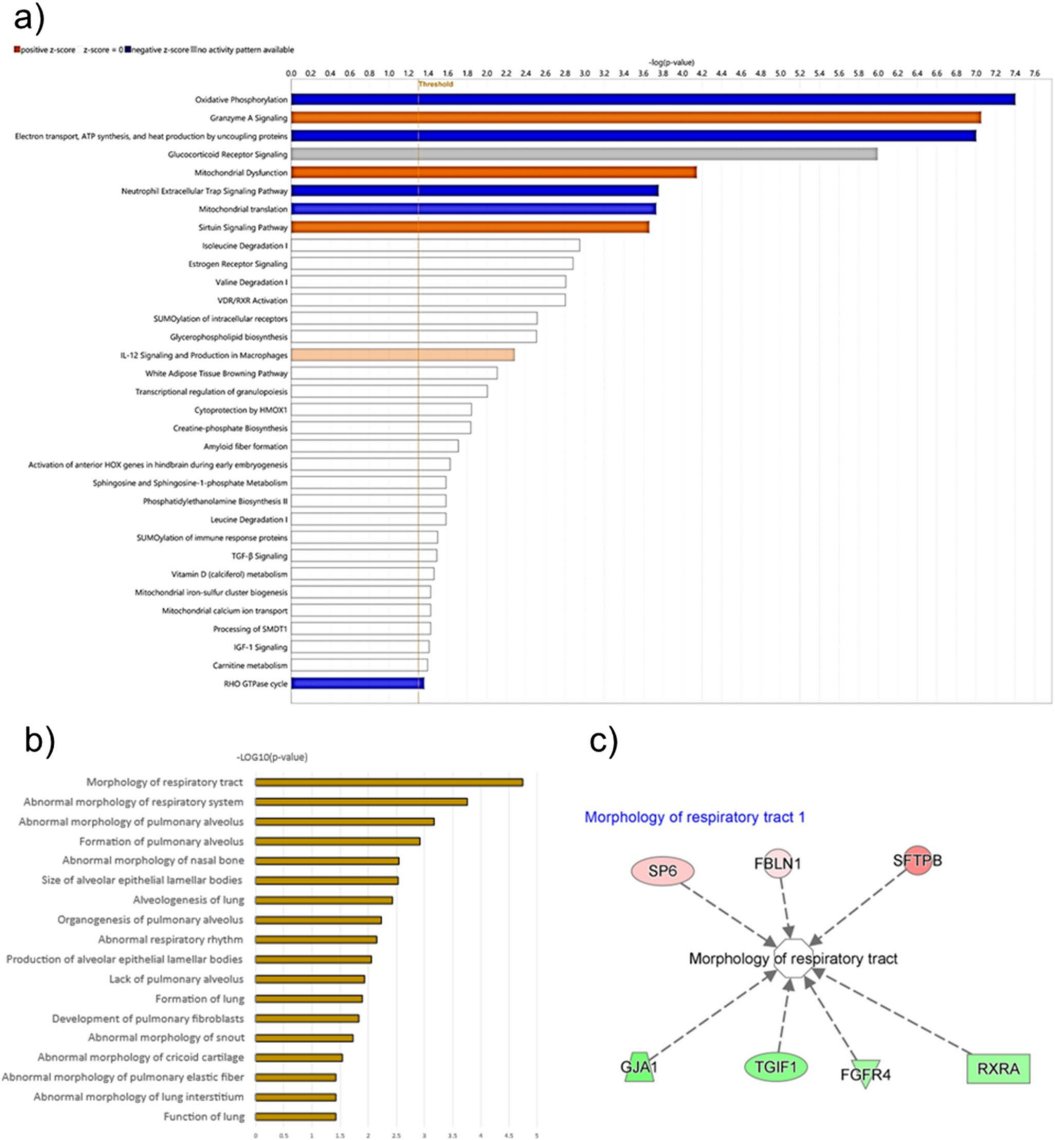
Pathway and Functional Analysis of Proteomic Data from Organoid Cultures. (**a**) Pathway analysis generated using Ingenuity Pathway Analysis (IPA) tool, showcasing the enriched biological pathways from proteomic data of organoids incubated in different conditions. Pathways are ranked by the -log(*p*-value), with the threshold set at *p* < 0.05, indicating the likelihood of the pathway being affected by the incubation condition. The length of the bar represents the significance level, with blue bars indicating a predicted activation and orange bars indicating a predicted inhibition of the pathway. (**b**) Functional analysis highlighting diseases and biological functions associated with differential protein expression. Functions are ranked by the -log(*p*-value), with the threshold set at *p* < 0.05. the length of the bar is representing the significance of the association. (**c**) Network analysis focusing on the "Morphology of respiratory tract" function, with key genes identified in the dataset that are predicted to affect this function based on the direction of their expression changes. Red nodes represent genes with increased expression, while green nodes represent genes with lowered expression in the Avatar incubator group compared to the normal condition group. Arrows indicate the direction of the predicted effect on the "Morphology of respiratory tract"

Utilizing the Ingenuity Pathway Analysis tool (IPA), our investigation into the regulated pathways revealed several key biological processes impacted by the incubation conditions (Fig. 8a). The most prominent pathways influenced included Mitochondrial Translation, Neutrophil Extracellular Trap Signalling Pathway, Mitochondrial Dysfunction, Glucocorticoid Receptor Signalling, as well as pathways related to Electron Transport, ATP Synthesis, and heat production by uncoupling proteins. Notably, pathways involved in Oxidative Phosphorylation and Granzyme A signalling were also found to be significantly regulated, indicating a broad range of cellular activities affected by the differential incubation.

Moreover, a detailed analysis was carried out to pinpoint diseases and functions that could be anticipated to alter in response to the observed molecular expression changes (Fig. 8b). This analysis identified 18 biological functions that were potentially impacted, with the top functions including the Formation of Pulmonary Alveolus, Abnormal Morphology of Pulmonary Alveolus, Abnormal Morphology of the Respiratory System, and Morphology of the Respiratory Tract.

Zooming in on the most significantly regulated function, the Morphology of the Respiratory tract, our data implicated seven genes that are involved in this biological process. SFTPB was observed to be affected with a substantial increase in expression (2.348 log2 FC), followed by SP6 and FBLN1, which also showed increased expression levels (1.057 and 0.532 log2 FC, respectively). Conversely, FGFR4, RXRA, TGIF1, and GJA1 all exhibited a decrease in expression, with log2 fold changes of -0.711, -0.757, -0.901, and -1.065, respectively. These findings suggest that the alterations in the expression of these specific genes are closely linked to the changes in the morphology of the respiratory tract observed in the organoid models under different incubation conditions.

## Discussion

We obtained alveolar organoids that have more complex morphology, increased branching and a better architectural representation of the distal lung by using a controlled microenvironment to generate alveolar lung organoids from induced pluripotent stem cells. Moreover, these organoids exhibit differential gene and protein expression profiles.

During early lung development, the embryo relies on diffusion to obtain oxygen and nutrients from the surrounding maternal tissues. The amniotic fluid provides a relatively stable and oxygenated environment. The pressure within the amniotic cavity is slightly higher than the maternal blood pressure, facilitating the diffusion of oxygen from the mother’s blood into the amniotic fluid. The oxygen concentration in the fetal lung is typically around 20–30 mm Hg: however, it gradually increases [6, 15–17]. This increase in oxygen concentration is due to several factors, including the growth of the lungs, the development of the alveoli (the tiny air sacs in the lungs where gas exchange takes place), and the production of surfactant. By the time the fetus is ready to be born, the oxygen concentration in the fetal lung is usually around 50–60 mm Hg, which is close to the oxygen concentration in the maternal lung [18]. This allows the fetus to transition to breathing air without complications. Therefore, in our system we maintained our organoids in a low oxygen concentration to mimic early embryonic development. Our protocol for generating the organoids from iPSCs starts with an initial exposure to hypoxia, followed by exposure to higher oxygen levels as the organoid matures. Oxygen regulates cell proliferation and maturation, and this is evident in our system where we observe higher expression of proteins that are responsible for lung maturation.

Soluble stem cell niche signals such as growth factors and cytokines are well documented and recognized for their role in regulating stem cell fate. Physical factors in the local cellular microenvironment including external mechanical forces, have strong influences on regulating stem cell fate[19]. Recently, the impact of insoluble biophysical cues like nanotopographical matrix mechanics and external mechanical forces have also been defined to play critical roles, with pressure being one of these key forces [20, 21]. The pressure within the developing lung is crucial in regulating lung maturation [22–24]. During the early stages of lung development, the pressure within the lung is lower than atmospheric pressure, which promotes the movement of fluid into the lung tissue. This fluid helps expand the lungs and creates space for alveoli formation [25]. As the lungs mature, the pressure within the lung increases, which helps to keep the alveoli inflated and allows for efficient gas exchange [26]. To mimic the physiological state, the system we used generates a positive pressure above ambient pressure that is transmitted to the cells. Since liquid, such as the culture medium, is incompressible, it effectively transfers the pressure to the cells. The increased branching, we observed in the cmO can be attributed to this phenomenon. Interestingly, previous studies have shown that mechanical stimuli can create an essential microenvironment that mimics the stem cell niche, which is confirmed in our study by the presence of cells marked for alveolar type II (ATII) cells and alveolar type I (ATI) cells, and basal cells alongside. Furthermore, correct cell positioning during developmental morphogenesis is critical and is suggested to be achieved through the appropriate establishment of mechanosignaling, which can be facilitated by pressure [27]. As a consequence of applying appropriate pressure, we observed a well-organized structural alignment of various cells in the alveolar lung organoid, giving it an architectural similarity to the alveolar space.

The controlled microenvironment not only influenced the architecture of the developed organoid but also regulated the transcriptomic level, with a notable upregulation of key genes of alveolar type I (ATI) and alveolar type II (ATII) cells in mature cmO compared to organoids grown under normal conditions. This indicates a positive effect of the controlled microenvironment. Moreover, proteomic analysis revealed an upregulation of proteins that play crucial roles in lung development. Three unique pathways were highlighted after pathway analysis was performed on the proteomic data, comparing the proteins expressed in the cmO to those in organoids grown under normal conditions. The top fourteen pathways were those involved in lung morphogenesis, development, or function.

The Gene Ontology (GO) terms "Cytostasis of lung cancer cell lines," "Progressive lung metastasis," "Function of lung," and "Abnormal morphology of lung interstitium" were significantly regulated. The two most prominently regulated proteins are ORM1 and SFTPB. ORM1 (orosomucoid 1) is an acute-phase protein known to regulate immune system activity. Although the precise role of ORM1 in lung development is not well understood, it has been identified as a biomarker for lung cancer [28], and has been shown to enhance cell proliferation. [29]. Surfactant protein B (SFTPB) is a major component of pulmonary surfactant and is secreted by both alveolar type II and club lung epithelial cells [30].

SFTPB plays a crucial role in lung development as it is the only surfactant protein strictly required for breathing. Its absence is associated with lethal respiratory failure in mice and humans [31]. Our data show that cells growing in the controlled microenvironment exhibit higher levels of factors that enhance the development and maturation of the organoids. It is worth noting that the two proteins downregulated in the cmO are ZC3H7A and RSPH10B2. The first protein, ZC3H7A, is a Zinc finger CCCH-type 7A that enables miRNA binding. The exact role of ZC3H7A in organogenesis, particularly lung development, is not well understood. However, it is known to regulate the biogenesis of miRNAs (MIR7-1, MIR16-2, and MIR29A). Notably, miR-29a plays a role in limiting proliferation in the late stages of lung development [32], suggesting that cmO might mimic an in utero regulatory network. miRNA 7 has been reported to be upregulated in COPD, although its exact role has not yet been identified [33]. The second protein, RSPH10B2, which is present in the cilia and associated with primary ciliary dyskinesia [34], is primarily expressed in the ciliated cells of the bronchus. Remarkably, this protein is also downregulated in the cmO, indicating the purity of the organoids. Our initial observations show that the cmO are pure alveolar organoids without cells from other parts of the respiratory tract. This was further confirmed by the absence of staining for cilia markers (Tubulin and MUC5AC).

To confirm that a controlled microenvironment does not induce a fibrotic response, we analyzed profibrotic markers in the cmO. No increased expression of Col1A1, Fibronectin, or KRT-17 was observed, indicating that cmO are promising for drug screening (data in Supplementary Figure, Fig S5).

Limitations of the current study include the important fact that although we have tried to mimic the microenvironment, we have only focused on two parameters and that too in static conditions in this proof-of-concept study. To truly replicate embryonic or fetal development, we need to have a system with sheer flow, potentially incorporating rotating cylinders and where pressure and oxygen can be controlled. Moreover, we have only demonstrated the results using one iPSC line. Further studies are warranted that use a system that is more complex and can include more parameters for microenvironmental control. These studies should also test this protocol in different iPSC lines. Also, a detailed step-by-step analysis of the effects of micro environment on differentiation needs to be elaborated at transcriptomics and proteomics level.

In conclusion, our successful generation of lung organoids from iPSCs, coupled with the enhanced development and maturation observed in a pressure and oxygen-controlled microenvironment, marks a significant leap in lung organoid research. This study not only underlines the role of microenvironmental factors in lung organoid formation but also positions these controlled in vitro models as potent tools for studying lung development and disease. The controlled microenvironment emerges as a critical determinant, offering an authentic platform for drug screening, disease modelling, and unravelling the intricacies of lung physiology. Our study sets a robust foundation, affirming the profound impact of microenvironmental control on the fidelity of in vitro lung organoid models.

## Material and Methods

### iPSCs Culture and Maintenance

iPSCs were cultured in StemFLEX media on rhLaminin521 coated plates. The cells were passaged with ReLeSR passaging reagent when 70–80% confluent.

Media was changed every alternate day. The cells were used for differentiation between passages 4 and 20, as described below. iPSCs were characterized by immunofluorescence staining of Oct3/4, Nanog, Sox-2.

iPSCs were reprogrammed from human foreskin fibroblasts (bought from ATCC) using the same reprogramming procedure as N7 iPSCs described before [35].

### Karyotyping for iPSCs

The CTG‐banding method used for Karyotyping, on twenty metaphase spreads as described before [36]. IPSCs were grown to 80% confluence and were treated for 2 h with 0.2 μg/ml colcemid (Thermo Scientific). Pre‐warmed hypotonic solution (KCl, 0.075 mol/l) was used to resuspend the cell pellets for 10 min at 37 °C, and freshly prepared, ice‐cold methanol‐acetic acid solution (3:1 in volume) was used to fix the cells; the cells were then mounted by dropping onto slides.

### iPSCs to Alveolar Lung Organoid Differentiation

iPSCs to lung organoid differentiation is based on an adapted version of the MERCK protocol 3dGRO™ Human Lung Organoid Culture System for differentiating iPSCs. Each media was aliquoted. Aliquots were frozen and thawed fresh before use.

iPSCs were cultured with StemFLEX media on rhLaminin521 in 6-well plates until they reached 80–90% confluence. A 6-well plate was coated with Matrigel (10 µL cold GFR Matrigel and 1 mL StemFLEX per well) and incubated at 37 °C for 1 h. iPSCs were dissociated with Accutase and resuspended in single cell passaging media (StemFLEX with 5 µM ROCK inhibitor). 1 × 10^6^ live cells were seeded onto the Matrigel coated plates and single cell passaging media was added to complete the well volume to 3 ml. 1.5 ml definitive endoderm induction medium (SCM302) was added to every well of iPSCs for days 1–3, with daily media changes. On day 3, a 6-well plate was coated with fibronectin (4 µg/mL) and stored at 4 °C overnight. Definitive endoderm cells were dissociated with Accutase and resuspended in AFE induction medium I (SCM305). 1 × 10^6^ definitive endoderm cells were seeded per well on to the fibronectin coated 6-well plate. On day 5 media was replaced with AFE induction medium II (SCM306). On days 6 and 7 media was replaced with 3dGRO lung organoid branching medium (SCM307). Once confluent on day 8, AFE cells were transferred to an ultra-low attachment 24-well plate as aggregates by scraping with a 2 ml serological pipette. Media was exchanged every second day with SCM307 by transferring the entire well into a 15 ml conical tube and allowing the cells to settle and accumulate at the bottom of the falcon. The supernatant was removed and the aggregates were gently resuspended and replated into the same well. By days 20–25, organoids with folded structures started to form. 4 to 6 of these folded organoids were embedded into one 24-well Transwell insert in a Matrigel sandwiches. 500 µl of SCM308 was added to both the basal and apical chambers of the inserts and media was exchanged every second day. The organoids started to develop branching structures around day 35 and were mostly mature after day 40. The culture can be maintained for longer. Parameters for the controlled microenvironment in Table 2.

**Table 2:** The parameters for the controlled microenvironment are as:

Cell type	Oxygen concentration	Pressure (PSI)
iPSCs	5%	0
iPSCs to DE	5%	0
DE to AFE	5%	2
AFE to Early LBO	5%	2
LBO to Mature LBO	10%	0.5

### Preparation of Histogel Cassettes for Paraffin Embedding

Epredia Histogel Specimen Processing Gel was preheated in a water bath set to 60 °C for 1 h. A Transwell insert with organoids was inverted and the exposed permeable membrane was cut out of the rim of the plastic insert carefully using a scalpel. A large drop of Histogel was added to a circular plastic mould, and the membrane was transferred using forceps and placed gently onto the mould with the insert side up. Another drop of Histogel was added on top, and the mould was placed on ice to solidify. After 15 min of incubation, the cast gel was removed from the mould and placed into a histology cassette. The cassette was placed into 4% PFA overnight at room temperature. The next day, the cassette was removed from 4% PFA and transferred to 70% ethanol before being further processed for Paraffin Embedding.

### Immunohistochemistry and Immunofluorescence

The organoids were paraffin embedded and cut into 5 µm thick sections. Slides were incubated in Xylol and deparaffinised, and tissue rehydration was performed in a descending ethanol concentration. The slides were stained with haematoxylin followed by two washing steps in water and counterstained in Eosin. Dehydration was performed in an ascending sequence of ethanol followed by a final Xylol treatment and the slides were mounted.

For immunofluorescence the organoids were fixed in 1% PFA and embedded in Tissue Tek (OCT solution) (Sakura Finetec, Japan). 7 µm thick cryosections were cut and were stained. Briefly, the cryosections were cut and washed with PBS twice, and incubated with blocking solution (150 mM maleimide + 1% BSA (in PBS)) for 1 h at room temperature. Washing twice in PBS, primary antibodies were added (antibodies are listed Table 3) to the section and then incubated overnight. After two washes with the PBS, secondary antibodies (antibodies are listed Table 4) were incubated for 2 h at room temperature, in the dark. After washing with PBS slides were stained with DAPI (Sigma-Aldrich, Cat No.: MBD0015, 2.5 µg/mL) and were fixed with fluorescence mounting medium (Dako, Cat No.: S302380-2). Imaging was performed using the CellVoyager™ CQ1 Benchtop High-Content Analysis System (Yokogawa, Japan) and the images were processed using CellPathfinder, High Content Analysis Software (Yokogawa, Japan). List of primary antibodies used are listed in Table 3 and of secondary antibodies Table 4.

**Table 3:** List of primary antibodies

Antibody	Company	Catalog Nr	Dilution	Stock concentration
E-Cadherin	BD Pharmingen	610182	1:50	250 µg/ml
SFTPC	Sigma	HPA010928	1:150	0.3 mg/ml
Podoplanin/PDPN	bio-techne	AF3670	1:100	0.2 mg/mL
HT1-56	Terrace Biotech	TB-29AHT1-56	1:100	n/a
KRT5	Biolegend	905903	1:800	0.5 mg/ml
HT2-280	Terrace Biotech	TB-27AHT2-280	1:150	n/a
KRT17	Sigma	HPA000453	1:100	0.08 mg/ml
Podoplanin/PDPN	Bio-Techne	AF3670	1:100	0.2 mg/mL
β-Tubulin	Novus Biologicals	MAB8527	1:750	0.5 mg/ml
Muc5AC	Invitrogen	MA5-12178	1:1000	0.2 mg/ml

**Table 4:** List of secondary antibodies

Antibody	Conjugate	Host	Company	Catalog Nr
Anti-mouse lgG	Alexa Fluor 488	Donkey	Invitrogen	A21202
Anti-rat lgG	Alexa Fluor 488	Donkey	Invitrogen	A21208
Anti-rabbit lgG	Alexa Fluor 568	Donkey	Invitrogen	A10042
Anti-sheep lgG	Alexa Fluor 647	Donkey	Invitrogen	A21448
Anti-chicken lgG	Alexa Fluor 647	Donkey	Jackson	703–605-155

### Antibody Conjugation

Lightning-Link Antibody Conjugation Kit (Abcam) was used according to manufacturer’s protocol for anti HT1-56 and anti HT2-280 antibodies (Terrace Biotech). 1 µl of modifier was added for every 10 µl of antibody and mixed gently with either Alexa Fluor 647 or Alexa Fluor 488 Conjugation mix and incubated for 15 min at room temperature in the dark. 1 µl of quencher was added to the mixture per 10 μl of antibody and after 5 min of incubation the linked antibodies were stored at 4 °C in the dark until use.

### Transmission Electron Microscopy

Organoids were grown in the Matrigel sandwich. Matrigel was removed using the Cell recovery solution (Corning, USA) following the protocol provided, 2.5% glutaraldehyde in 0.15 M HEPES buffer (707 mOsm, pH 7.4) was used to fix the organoids; later the organoids were stored in fixative solution at 4 °C. Further, the same fixative was used for rinsing; the organoids were postfixed for 1 h with a solution of 0.1 M sodium cacodylate buffer with 1% solution of osmium teratoxide (369 mOsm, pH 7.4). The organoids were rinsed with 0.05 M maleate buffer (pH 5.0), and postfixed in 0.05 M maleate buffer containing 0.5% uranyl acetate for 1 h. The organoids were dehydrated in consequently increasing concentrations of ethanol (70%, 80%, 96%, 100%) and were incubated overnight in ethanol and epon (1:1) mixture. Next morning, cells were embedded in epon resin and incubated at 60 °C to polymerize for 6 days. Reichert-Jung Ultracut E microtome was used to cut ultrathin Sects. (70 nm); the sections were put on formyar coated 2 mm × 1 mm single slot copper grids and double stained with 1% uranyl acetate and 3% lead citrate. Philips EM 400 transmission electron microscope, using Morada digital camera and iTEM/cell^F program was used for obtaining images [37, 38]

### Relative Gene Expression by RT-qPCR

RNA was extracted from organoids using the NucleoSpin RNA extraction kit (Macherey–Nagel, Germany) according to manufacturer’s protocol. Reverse transcription was done using the Omniscript RT kit (Qiagen) following the manufacturer’s protocol. cDNA amplification was done with Fast SYBR Green Master Mix (Applied Biosystems,USA) on an Applied Biosystems 7500 Real-Time Fast PCR (Life Technologies,USA) machine. Analysis of relative mRNA expression was done using the ΔΔCt method, normalizing against the housekeeping gene β-actin. List of primers used (Table 5).

**Table 5:** The list of used primers

Primers	Sequence
hNKX2.1_F	GGA CGT GAG CAA GAA CAT G
hNKX2.1_R	TCG CTC CAG CTC GTA CAC C
hFOXA2_F	GGG AGC GGT GAA GAT GGA
hFOXA2_R	TCA TGT TGC TCA CGG AGG AGT A
hSFTPA_F	CTG TCC CAA GGA ATC CAG AG
hSFTPA _R	CCG TCT GAG TAG CGG AAG TC
hSFTPB_F	CAA TGA TTC CAA GGG TGC G
hSFTPB_R	CAG CAA TCC TCC TGT CGG C
hSFTPC_F	TCC AGA GAG CAT CCC CAG TC
hSFTPC_R	GGC TTC CAC TGA CCC TGC
hSFTPD_F	AGG AG C A AA GGG AG A A AG TGG G
hSFTPD_R	CAG CT GTG C C TC CGT AA A T GG
ABCA3_F	AGA TGT AGC GGA CGA GAG GA
ABCA3_R	GCT GCT CGT ACA CCT TGG AG
LAMP3_F	AAG ATG ACC ACT TTG GAA ATG TG
LAMP3_R	GAT GGC CCC AAT CAC AGG AA
Β Actin_F	CAA GA G A TG GCC AC G G CT GCT
Β Actin_F	TCC TT C T GC AT C C TG TCG GC A
Col1A1_F	ATG TTC AGC TTT GTG GAC CTC
Col1A1_R	CTG TAC GCA GGT GAT TGG TG
Fibronectin_F	ACA ACA CCG AGG TGA CTG AGA C
Fibronectin_R	GGA CAC AAC GAT GCT TCC TGA G
KRT-17_F	ATC CTG CTG GAT GTG AAG ACG C
KRT-17_R	TCC ACA ATG GTA CGC ACC TGA C

### Flow Cytometry

For flow cytometry, adherent cells (definitive endoderm, anterior foregut endoderm) were released by TrypLE, for early lung organoids floating cells were collected and centrifuged and for mature organoids the Matrigel was dissolved using the Cell recovery solution (Corning, USA) following the protocol provided. Early and mature organoids were dissociated into single cells by incubation with Accutase (Sigma-Aldrich, A6964-500ML) for 15 min at 37 °C, followed by vigorous pipetting. After washing, the cells were blocked with FcR blocking reagent (Miltenyi Biotec, 130–059-901) on ice for 20 min in the dark. Then, cells were stained in the dark for 20 min with the relevant extracellular antibody mix and Zombie Violet Fixable viability dye (BioLegend, 423,113) on ice, followed by two washes with the cell staining buffer. Cells were fixed in 1% PFA for 10 min on ice in the dark and washed two times. Cells were permeabilized and stained with a permeabilization buffer (Thermo Scientific, 00–8333-56) with the relevant intracellular antibody mix and incubated in the dark for 30 min at room temperature. The cells were then washed two times with cell staining buffer. Lastly, the fixed and stained cells were resuspended in cell staining buffer with 5% FBS and stored in the dark, protected from light. Flow cytometry was performed on different instruments depending on the experiment: Cytek Aurora (Cytek Bio) for the trilineage differentiation or ImageStreamX (Amnis) for the differentiating cells and organoids. ImageStreamX, an imaging flow cytometer, combines the high throughput performance of flow cytometry with the imagery and functional insights of microscopy, it is multispectral including 12 channels, and supported by a sophisticated software Ideas that analyses the flow cytometry, microscopy data and identifies the spatial location [39]. For early lung organoids and mature lung organoids four transwell inserts were pooled to obtain high cell numbers. The experiments were repeated twice and the data is presented as mean ± SEM. For Cytek Aurora the data was analysed in Flowjo analysis software (BD biosciences, USA). Gating strategy (Figure S3) and the antibody details (Table S2) are provided in the supplemental section.

### Proteomics

#### Sample Preparation and Instrumentation Setup

For each experimental condition, three samples (n = 3 per condition) were prepared and analyzed. The samples were resuspended in a solution containing 8 M urea and 100 mM Tris (pH 8). Protein concentration was determined using the Qubit Protein Assay (Invitrogen by Life Technologies, Zug, Switzerland). A 10 µg aliquot of the protein was then subjected to reduction, alkylation, and digestion. Digestion was first carried out using LysC for 2 h at 37 °C, followed by overnight digestion with trypsin at room temperature as described earlier [40].

The resulting digests were analyzed using nano-liquid chromatography coupled with tandem mass spectrometry (LC–MS/MS). This was performed on a Nano Elute2 system linked to a timsTOF HT (Bruker Daltonics, Bremen, Germany) through a CaptiveSpray source (Bruker, Bremen, Germany). Instrument settings included an end-plate offset of 500 V, a drying temperature of 200 °C, and a capillary voltage set at 1.6 kV. For chromatographic separation, a 5 µL (500 ng) sample of the protein digest was first loaded onto a pre-column (C18 PepMap 100, 5 µm, 100 Å, 300 µm i.d. x 5 mm length, ThermoFisher). The sample was then eluted in back-flush mode onto a 10 cm analytical column (C18 Reprosil AQ, 1.9 µm, 75 µm, Bruker Daltonics, Bremen, Germany) using a 30-min gradient ranging from 5 to 40% acetonitrile in water with 0.1% formic acid, at a flow rate of 500 nL/min.

The timsTOF HT instrument was operated in either Data-Dependent Acquisition (DDA) or Data-Independent Acquisition (DIA) mode using the Parallel Acquisition Serial Fragmentation (PASEF) technique. For DDA, the mass range was set between 100 and 1700 m/z, with 8 PASEF scans between 0.75 and 1.35 V s/cm^2^. The accumulation and ramp times were both set to 100 ms. Fragmentation occurred at 15,000 arbitrary units (au), with peptides (up to a charge state of 5) fragmented using collision-induced dissociation (CID) with an energy spread ranging from 20 to 75 eV.

The dia-PASEF acquisition method was configured with 47 isolation windows, each 26 m/z wide, with a 1 m/z overlap between adjacent windows. These isolation windows corresponded to an ion mobility range of 0.7 to 1.45 V s/cm^2^. Both TIMS accumulation and separation times were set at 100 ms.

DDA data were employed to generate a spectral library using FragPipe [40], version 20.0, with the following parameters: the human database from SwissProt (April 2023 release) was supplemented with common contaminants. Mass tolerances were set to ± 20 ppm for MS1 and ± 0.05 Da for MS2. Trypsin served as the cleavage enzyme, allowing up to 3 missed cleavages. Dynamic modifications included oxidation on methionine and protein N-terminal acetylation, while carbamidomethylation of cysteines was considered a fixed modification. The minimum number of matched fragments was set to 5. PSM validation was performed using the default Percolator settings, with MSBooster enabled.

For DIA data analysis, Spectronaut (Biognosys), version 18.4.231011.55695, was utilized in peptide-centric mode with the spectral library mentioned above. Factory settings were applied, and single-hit proteins were excluded.

Data Analysis: Potential contaminants were filtered out before conducting differential expression and subsequent analyses. Protein intensity missing values were imputed as follows: if there was at most one detection in a replicate group, the remaining missing values were imputed by randomly drawing from a Gaussian distribution, with a width of 0.3 times the sample standard deviation and shifted left by 2.5 times the sample standard deviation from the sample mean. All other missing values were replaced using the Maximum Likelihood Estimation method. Differential expression testing was conducted using the moderated t-test, with multiple test correction performed via the Benjamini-Hochberg [BH] adjustment. Significance criteria, utilizing 20 imputation cycles, were applied as described with a minimum absolute log2 fold change of 1 and a maximum adjusted p-value of 0.05 (the latter achievable at asymptotically high log2 fold changes).

#### Generation of Heatmaps and Hierarchical Clustering Analysis

Hierarchical clustering and heatmap visualizations were constructed employing the “heatmap2” function available within the R package “Gene-E.” Initially, a pairwise correlation matrix was computed for the items using the Pearson correlation coefficient to quantify linear relationships. This correlation matrix was subsequently transformed into a distance matrix through a dissimilarity measure, which formed the basis for the clustering algorithm. Clustering was executed using the average linkage method, which calculates the average pairwise distance between clusters to build the dendrogram. The resulting heatmaps were generated to visually represent the hierarchical organization and correlation patterns among the items.

#### Quantitative Visualization through Volcano Plot Construction

In this investigation, we utilized the R programming environment (version 4.3.3, “Angel Food Cake”) for the construction of a volcano plot, a quantitative method for the visualization of differential protein expression. The plot was generated using the EnhancedVolcano package (Release 3.18), with data input comprising log2 fold changes (log2FC) and associated p-values from the statistical analysis. The focus was on identifying proteins demonstrating statistically significant expression differences, with a stringent p-value threshold of 0.05 applied. The volcano plot’s x-axis depicts the log2FC of protein abundance between experimental conditions, while the y-axis represents the negative logarithm (base 10) of the computed p-values. This dual-axis representation facilitated the identification and interpretation of proteins exhibiting significant expression alterations. Protein identifiers were annotated on the plot using connectors for enhanced clarity. A horizontal dashed line demarcates the threshold for statistical significance, set at a p-value of 0.05, delineating proteins that surpass this criterion. Additionally, vertical dashed lines were incorporated to signify the cutoff for fold changes, specifically at 0.5 log2 fold change, thereby highlighting proteins with biologically meaningful expression shifts.

#### Principal Component Analysis (PCA) for Multivariate Expression Profiling

For the multivariate analysis of log2 fold change in mRNA and microRNA expression data between patient and control groups, principal component analysis (PCA) was performed using the ‘prcomp’ function in R (R Core Team, 2016). The dimensionality reduction was visualized in three-dimensional space using the rgl and scatterplot3d R packages. To enhance numerical precision, the PCA was conducted via singular value decomposition of the pre-processed data matrix, which was both centred and scaled.

#### Z-Score Computation for Canonical Pathway Activity Inference

To infer the modulation of canonical pathways and functional endpoints based on differential gene expression, we computed the pathway activity Z-score using Ingenuity Pathway Analysis (IPA). This metric allowed us to determine whether the activity of specific biological pathways was upregulated or downregulated in response to the experimental conditions.

#### Upstream Regulator Enrichment Analysis via IPA

We conducted an enrichment analysis for upstream regulators using the upstream pathway analysis module of Ingenuity Pathway Analysis (IPA), with data reflecting the state as of December 29, 2023. The analysis involved calculating overlapping P values via Fisher’s exact test, assessing the statistical significance of overlaps between known regulatory targets and the experimentally derived set of differentially expressed genes.

#### Diseases & Functional Annotation through IPA Functional Analysis

This study incorporated a comprehensive Diseases & Functions analysis using Ingenuity Pathway Analysis (IPA) to elucidate potential molecular associations with known disease states and biological functions. The IPA Functional Analysis leverages the curated biological interactions from the Ingenuity Knowledge Base to systematically organize and interpret the biological relevance of our dataset. This analysis aimed to provide a high-level overview of the functional landscape pertinent to our data, with a particular emphasis on lung-related processes. By systematically probing and identifying the most relevant biological functions and diseases, this analysis facilitated a deeper understanding of the underlying molecular mechanisms and their implications for pulmonary biology.

### Statistical Analysis

Data is presented as mean ± SEM, for all experiments. Graph Prism 10 software (Graph Pad software Inc., San Diego, CA, USA) was used for designing of graphs and statistical analysis. A parametric non-paired t-test was used, and p˃0.05* was considered significant.

## Supplementary Information

Below is the link to the electronic supplementary material.

Supplementary file1 (DOCX 35 KB)

Supplementary file2 (DOCX 36 KB)

## Data Availability

All data supporting the findings of this study are available within the paper and its Supplementary Information.

## References

[1] Altorki, N. K., et al. (2019). The lung microenvironment: An important regulator of tumour growth and metastasis. *Nature Reviews Cancer,**19*(1), 9–31. 30532012 10.1038/s41568-018-0081-9 PMC6749995

[2] Schittny, J. C. (2017). Development of the lung. *Cell and Tissue Research,**367*(3), 427–444. 28144783 10.1007/s00441-016-2545-0 PMC5320013

[3] Bain, C. C., & MacDonald, A. S. (2022). The impact of the lung environment on macrophage development, activation and function: Diversity in the face of adversity. *Mucosal Immunology,**15*(2), 223–234. 35017701 10.1038/s41385-021-00480-w PMC8749355

[4] Strunz, M., et al. (2020). Alveolar regeneration through a Krt8+ transitional stem cell state that persists in human lung fibrosis. *Nature Communications,**11*(1), 3559. 32678092 10.1038/s41467-020-17358-3 PMC7366678

[5] Stamati, K., Mudera, V., & Cheema, U. (2011). Evolution of oxygen utilization in multicellular organisms and implications for cell signalling in tissue engineering. *Journal of Tissue Engineering,**2*(1), 2041731411432365. 22292107 10.1177/2041731411432365 PMC3258841

[6] Stevens, R. P., et al. (2023). Got oxygen? Studies on mesenchymal cell hypoxia inducible factor-1α in lung development. *American Journal of Respiratory Cell and Molecular Biology,**69*(4), 380–382. 37478332 10.1165/rcmb.2023-0247ED PMC10557915

[7] Tingay, D. G., et al. (2023). Inflating pressure and not expiratory pressure initiates lung injury at birth in preterm lambs. *American Journal of Respiratory and Critical Care Medicine,**208*(5), 589–599. 37276583 10.1164/rccm.202301-0104OC

[8] Metzger, R. J., et al. (2008). The branching programme of mouse lung development. *Nature,**453*(7196), 745–750. 18463632 10.1038/nature07005 PMC2892995

[9] Conway, R. F., et al. (2020). Understanding human lung development through in vitro model systems. *BioEssays,**42*(6), 2000006. 10.1002/bies.202000006 PMC7433239 32310312

[10] Shibuya, S., et al. (2021). In vitro models of fetal lung development to enhance research into congenital lung diseases. *Pediatric Surgery International,**37*(5), 561–568. 33787982 10.1007/s00383-021-04864-8 PMC8026475

[11] Shi, Y., et al. (2017). Induced pluripotent stem cell technology: A decade of progress. *Nature Reviews Drug Discovery,**16*(2), 115–130. 27980341 10.1038/nrd.2016.245 PMC6416143

[12] Zhao, Z., et al. (2022). Organoids. *Nature Reviews Methods Primers,**2*(1), 94. 37325195 10.1038/s43586-022-00174-y PMC10270325

[13] Yang, S., et al. (2023). Organoids: The current status and biomedical applications. *MedComm,**4*(3), e274. 37215622 10.1002/mco2.274 PMC10192887

[14] Calà, G., et al. (2023). Primary human organoids models: Current progress and key milestones. *Frontiers in Bioengineering and Biotechnology,**11*, 1058970. 36959902 10.3389/fbioe.2023.1058970 PMC10029057

[15] Browne, V. A., et al. (2015). Uterine artery blood flow, fetal hypoxia and fetal growth. *Philosophical Transactions of the Royal Society of London. Series B, Biological sciences,**370*(1663), 20140068. 25602072 10.1098/rstb.2014.0068 PMC4305169

[16] Carter, A. M. (2015). Placental gas exchange and the oxygen supply to the fetus. *Comprehensive Physiology,**5*(3), 1381–1403. 26140722 10.1002/cphy.c140073

[17] Gao, Y., et al. (2016). Unique aspects of the developing lung circulation: Structural development and regulation of vasomotor tone. *Pulm Circ,**6*(4), 407–425. 27942377 10.1086/688890 PMC5130074

[18] Jobe, A. H., & Abman, S. H. (2019). Bronchopulmonary dysplasia: A continuum of lung disease from the fetus to the adult. *American Journal of Respiratory and Critical Care Medicine,**200*(6), 659–660. 31091958 10.1164/rccm.201904-0875ED PMC6775887

[19] Sun, Y., Chen, C. S., & Fu, J. (2012). Forcing stem cells to behave: A biophysical perspective of the cellular microenvironment. *Annual Review of Biophysics,**41*(1), 519–542. 22404680 10.1146/annurev-biophys-042910-155306 PMC4123632

[20] Orr, A. W., et al. (2006). Mechanisms of mechanotransduction. *Developmental Cell,**10*(1), 11–20. 16399074 10.1016/j.devcel.2005.12.006

[21] Vogel, V., & Sheetz, M. (2006). Local force and geometry sensing regulate cell functions. *Nature Reviews Molecular Cell Biology,**7*(4), 265–275. 16607289 10.1038/nrm1890

[22] Nelson, C. M., et al. (2017). Microfluidic chest cavities reveal that transmural pressure controls the rate of lung development. *Development,**144*(23), 4328–4335. 29084801 10.1242/dev.154823 PMC5769635

[23] Warburton, D., et al. (2010) *Chapter Three - *Lung* Organogenesis* (pp. 73–158). In Current Topics in Developmental Biology, P. Koopman, Editor, Academic Press. 10.1016/S0070-2153(10)90003-3 PMC3340128 20691848

[24] Miller, A. A., Hooper, S. B., & Harding, R. (1993). Role of fetal breathing movements in control of fetal lung distension. *Journal of Applied Physiology,**75*(6), 2711–2717. 8125894 10.1152/jappl.1993.75.6.2711

[25] Davis, R. P., & Mychaliska, G. B. (2013). Neonatal pulmonary physiology. *Seminars in Pediatric Surgery,**22*(4), 179–184. 24331091 10.1053/j.sempedsurg.2013.10.005

[26] Plosa, E., & Guttentag, S. H. (2018). 42 - Lung Development. In C. A. Gleason & S. E. Juul (Eds.), *Avery’s Diseases of the Newborn (Tenth Edition)* (pp. 586-599.e2). Elsevier.

[27] Argentati, C., et al. (2019). Insight into Mechanobiology: How Stem Cells Feel Mechanical Forces and Orchestrate Biological Functions. *International Journal of Molecular Sciences,**20*(21), 5337. 31717803 10.3390/ijms20215337 PMC6862138

[28] Ye, X., et al. (2020). Dramatically changed immune-related molecules as early diagnostic biomarkers of non-small cell lung cancer. *FEBS Journal,**287*(4), 783–799. 31482685 10.1111/febs.15051

[29] Yu, G., et al. (2023). ORM1 promotes tumor progression of kidney renal clear cell carcinoma (KIRC) through CALR-mediated apoptosis. *Scientific Reports,**13*(1), 15687. 37735575 10.1038/s41598-023-42962-w PMC10514263

[30] Weaver, T. E., & Conkright, J. J. (2001). Function of surfactant proteins B and C. *Annual Review of Physiology,**63*(1), 555–578. 11181967 10.1146/annurev.physiol.63.1.555

[31] Melton, K. R., et al. (2003). SP-B deficiency causes respiratory failure in adult mice. *American Journal of Physiology-Lung Cellular and Molecular Physiology,**285*(3), L543–L549. 12639841 10.1152/ajplung.00011.2003

[32] Bhaskaran, M., et al. (2009). MicroRNA-127 modulates fetal lung development. *Physiological Genomics,**37*(3), 268–278. 19439715 10.1152/physiolgenomics.90268.2008 PMC2685501

[33] Akbas, F., et al. (2012). Analysis of serum micro-rnas as potential biomarker in chronic obstructive pulmonary disease. *Experimental Lung Research,**38*(6), 286–294. 22686440 10.3109/01902148.2012.689088

[34] Coan, M., et al. (2018). Exploring the role of fallopian ciliated cells in the pathogenesis of high-grade serous ovarian cancer. *International Journal of Molecular Sciences,**19*(9), 2512. 30149579 10.3390/ijms19092512 PMC6163198

[35] Grad, I., et al. (2011). NANOG priming before full reprogramming may generate germ cell tumours. *European Cells and Materials,**22*, 258–74. 22071697 10.22203/ecm.v022a20

[36] Hibaoui, Y., et al. (2014). Modelling and rescuing neurodevelopmental defect of Down syndrome using induced pluripotent stem cells from monozygotic twins discordant for trisomy 21. *EMBO Molecular Medicine,**6*(2), 259–277. 24375627 10.1002/emmm.201302848 PMC3927959

[37] Khan, P., et al. (2018). Culture of human alveolar epithelial type II cells by sprouting. *Respiratory Research,**19*(1), 204. 30340591 10.1186/s12931-018-0906-9 PMC6195695

[38] Tamo, L., et al. (2018). Generation of an alveolar epithelial type II cell line from induced pluripotent stem cells. *American Journal of Physiology. Lung Cellular and Molecular Physiology,**315*(6), L921–L932. 30211653 10.1152/ajplung.00357.2017

[39] Clark, R. T. (2015). Imaging flow cytometry enhances particle detection sensitivity for extracellular vesicle analysis. *Nature Methods,**12*(4), i–ii.

[40] Braga-Lagache, S., et al. (2016). Robust label-free, quantitative profiling of circulating plasma microparticle (MP) associated proteins. *Molecular and Cellular Proteomics,**15*(12), 3640–3652. 27738094 10.1074/mcp.M116.060491 PMC5141277

