# Supplementary material for: Influence of Microenvironmental Orchestration on Multicellular Lung Alveolar Organoid Development from Human Induced Pluripotent Stem Cells
Source: Stem Cell Rev Rep. 2024 Oct 17;21(1):254–75. doi: 10.1007/s12015-024-10789-1 (PMC11762634; doi:10.1007/s12015-024-10789-1)
Supplement: Supplementary file 1 — Supplementary file1 (DOCX 35 KB) [file 12015_2024_10789_MOESM1_ESM.docx]

**Figure legend for Supp figures**

**Figure S1:** Immunofluroscence of iPSCs for (A) Oct3/4, (B) Nanog, (C) Representative karyogram of the iPSCs no Chromosomal abrasion was observed.

**Figure S2:** Flow cytometery for triliniage differentiation (A)CD140b (mesoderm) (B) CXCR4 (endoderm) (C) Pax6 (ectoderm) (D) VE cadherin (Mesoderm) (E) SOX17 (endoderm) (F) Sox2 (ectoderm). Representative immunofluroscence images (G) CXCR4 (definitive endoderm) for normal and for cmO (H) Epcam (Anterior foregut endoderm) for normal (I) and for cmO (J).

**Figure S3:** Gating strategy of FACS: For each sample, at least 10,000 live, single events were recorded and gating was done using the IDEAS software by AMNIS, the fcs files were used to analyse the Image Stream data and to calculate the compensation. Debris was excluded from single cells. Live cells were differentiated by Live/Dead staining. Single stained beads were used for each fluorophore to decide on the gates along with unstained samples of each cell type. Since laser imaging flow cytometry directly measures spatial variation in the brightfield, darkfield and fluorescence images, in a well-focused brightfield image, the cell boundaries create a sharp intensity change and hence a high gradient value. The in-focus sub-population are then defined and made with a high-pass gating of the gradient histogram. After defining the in-focus cellular events, the *focus* sub-population is further filtered to select single cells. Based on morphological features obtained from the brightfield image produce a 2D scatter plot of aspect ratio versus area. A single-cell sub-population is defined from the dense cluster of events with high aspect ratio (tending to circular shape) and intermediate area (lying above a band of smaller objects corresponding to debris, and below higher points representing images containing multiple cells). After gating to define the single cell, in focus population the lasers are defined and the images are analysed using the IDEAS software as defined before [1].

**Definitive Endoderm (DE): 3a. Definitive endoderm unstained (normal) 3b.** Definitive endoderm stained (normal) for CXCR4+ C-Kit+& NKX2-1+ population was gated for single cells in live cells. Similarly for the cmO **3c**.definitive endoderm unstained (cmO); **3d**.definitive endoderm stained (cmO). **Anterior Foregut Endoderm (AFE): 3e.** Unstained (normal), **3f.** AFE (normal) NKX2-1+, EpCAM+, Sox9+, Pax9+ positive cells were gated for single cells in live cells. **3g.** AFE (cmO) unstained, **3h**. AFE (stained). **Early Alveolar Lung Organoids (Organoids in Suspension) 3i.** ELBO Unstained (normal)**, 3j.** ELBO stained (normal) NKX2-1+, EpCAM+ and Sox9+ population were gated for under live cells; SFTPB+ and SFTPC+ populations were gated under EPCAM+ cells. **3k.** ELBO unstained (cmO), **3l.** ELBO stained (cmO). **Mature Alveolar Lung Organoids (Organoids in Matrigel Sandwich) 3m.** unstained (normal)**, 3n.** stained (normal) NKX2-1+, EpCAM+, and Sox9+ population were gated under live cells; SFTPB+ and SFTPC+ populations were gated under EPCAM+ cells. **3o** unstained (cmO), **3p** mature organoids stained.

**Figure S4:** Representative images of all the cell types stained with different antibodies is shown along with a bright field image of the single cells (Images generated by IDEAS software).

**Figure S5:** Relative mRNA expression of markers of fibrosis (A) COL1A1 (0.50±0.18) (B) Fibronectin (0.83±0.12) (C) KRT17 (0.96±0.04) in cmO. The values are normalized to the organoids growing in normal condition. Data represented as 2∆∆Ct values.

References:

1. Rees, P., et al., *Imaging flow cytometry: a primer.* Nat Rev Methods Primers, 2022. **2**.
