# Supplementary material for: Influence of Microenvironmental Orchestration on Multicellular Lung Alveolar Organoid Development from Human Induced Pluripotent Stem Cells
Source: Stem Cell Rev Rep. 2024 Oct 17;21(1):254–75. doi: 10.1007/s12015-024-10789-1 (PMC11762634; doi:10.1007/s12015-024-10789-1)
Supplement: Supplementary file 2 — Supplementary file2 (DOCX 36 KB) [file 12015_2024_10789_MOESM2_ESM.docx]

| **Cell Type** | **Marker** | **Fluorophore** | **Concentration (Antibody/cells)** | **Catalog Number** | **Manufacturer** |
| --- | --- | --- | --- | --- | --- |
| Mesoderm | CD144 (VE-Cadherin) | FITC | 1 µl to 50 µl | 130-123-932 | Miltenyi Biotec |
| Mesoderm | CD140b | APC | 1 µl to 50 µl | 130-121-128 | Miltenyi Biotec |
| Endoderm | CXCR4 (CD184) | APC | 1 µl to 50 µl | 130-120-708 | Miltenyi Biotec |
| Endoderm | Sox17 | FITC | 1 µl to 50 µl | 130-111-147 | Miltenyi Biotec |
| Ectoderm | Pax6 | APC | 1 µl to 50 µl | 130-123-328 | Miltenyi Biotec |
| Ectoderm | Sox2 | FITC | 1 µl to 50 µl | 130-120-790 | Miltenyi Biotec |

**Supp Data**

1. List of Antibodies for Trilineage differentiation
2. List of antibodies for Flow cytometery

| **Cell Type/Organoid** | **Marker** | **Fluorophore** | **Concentration (Antibody/cells)** | **Catalog Number** | **Manufacturer** |
| --- | --- | --- | --- | --- | --- |
| Definitive Endoderm | CXCR4 (CD184) | PE | 5 µl to 100 µl | 306505 | BioLegend |
| Definitive Endoderm | C-Kit (CD117) | Alexa Fluor 647 | 5 µl to 100 µl | 313235 | BioLegend |
| Definitive Endoderm | Nkx2-1 (TTF-1) | Alexa Fluor 594 | 5 µl to 100 µl | NBP2-34544 | Novus Biologicals |
| Anterior Foregut Endoderm | Sox2 | PE | 5 µl to 100 µl | 656103 | BioLegend |
| Anterior Foregut Endoderm | Pax9 | FITC | 1 µl to 100 µl | 324212 | Abcam |
| §Anterior Foregut Endoderm | EpCAM (CD326) | Alexa Fluor 647 | 1 µl to 500 µl | NBP2-34544 | Novus Biologicals |
| Anterior Foregut Endoderm | Nkx2-1 (TTF-1) | Alexa Fluor 594 | 5 µl to 100 µl | 324212 | Abcam |
| Alveolar Organoids | Nkx2-1 (TTF-1) | Alexa Fluor 594 | 5 µl to 100 µl | 324212 | Abcam |
| Alveolar Organoids | EpCAM (CD326) | Alexa Fluor 647 | 1 µl to 500 µl | NBP2-34544 | Novus Biologicals |
| Alveolar Organoids | Sox9 | FITC | 2 µl to 100 µl | sc-166505 | Santa Cruz |
| Alveolar Organoids | SFTPC | Alexa Fluor 546 | 2 µl to 100 µl | sc-518029 | Santa Cruz |
| Alveolar Organoids | SFTPB | Alexa Fluor 680 | 2 µl to 100 µl | sc-133143 | Santa Cruz |
| Live/Dead Staining | Zombie Violet | Live/Dead | 1 µl to 1000 µl | 423113 | BioLegend |

C) Laser information for image stream used for flow cytometery

| Wavelength | Intensity (mW) |
| --- | --- |
| 405nm | 40.00 |
| 488nm | 100.00 |
| 561nm | 50.00 |
| 642nm | 50.00 |
| 785nm | 1.00 |
